# TopCas: Topology‐Gated Cas12a via DNA‐RNA Chimeric Circular crRNA for Amplification‐Free Nucleic Acid Detection and Conditional Gene Editing

**DOI:** 10.1002/advs.75046

**Published:** 2026-03-30

**Authors:** Shun Zhang, Wen Sun, Ting Xiao, Yali Wang, Xianlan Wu, Huiyou Chen, Ming Chen, Jun Zhang

**Affiliations:** ^1^ Department of Clinical Laboratory Medicine Southwest Hospital Third Military Medical University (Army Medical University) Chongqing P. R. China; ^2^ Department Key Laboratory of Bio‐Resource and Eco‐environment of Ministry of Education, College of Life Sciences Sichuan University Chengdu Sichuan P. R. China

**Keywords:** amplification‐Free, chimeric crRNA, circular crRNA, CRISPR Cas12a, topology‐gated

## Abstract

Controlled activation of CRISPR‐Cas12a is critical for achieving conditional gene editing and molecular diagnostics. As an indispensable component for forming an active complex, CRISPR RNA (crRNA) represents a key route to regulate Cas12a activity. Here, we establish TopCas (Topology‐gated Cas12a via DNA‐RNA Chimeric Circular crRNA) as a platform for preamplification‐free nucleic acid detection and conditional gene editing. Within TopCas, the circular crRNA sterically constrains Cas12a's nuclease activity until target‐activated complexes trans‐cleave the DNA segment of the chimeric crRNA, converting the circular guide into its linear form and initiating an autocatalytic cascade that culminates in fluorophore release and signal amplification. By the same mechanism, the system conditionally activates Cas12a's gene‐editing function (cis‐cleavage) exclusively in the presence of specific nucleic acid targets (e.g., viral DNA or RNA). We demonstrate that TopCas affords high specificity and sensitivity in nucleic acid detection, supports accurate detection in clinical viral nucleic acid samples, and shows potential for in vivo real‐time molecular imaging, while also demonstrating the feasibility of conditional gene editing. This innovative chimeric circular crRNA‐Cas12a system not only provides a new tool for precise disease diagnostics but also offers a promising strategy for personalized therapeutic intervention.

## Graphical abstract

The TopCas platform utilizes an engineered DNA‐RNA chimeric circular crRNA to establish topology‐gated control over CRISPR‐Cas12a activity, enabling target‐triggered self‐catalytic amplification (preamplification‐free). This system seamlessly integrates highly sensitive molecular diagnostics with conditionally controlled gene editing, demonstrating significant potential for clinical detection and precision gene therapy applications.

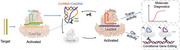

## 1 Introduction

The CRISPR–Cas systems have revolutionized the fields of genome engineering and molecular diagnostics by virtue of their precisely engineered nucleic acid‐targeting capabilities [1, 2, 3]. In particular, Cas12a (Cpf1) combines a compact single‐RNA architecture with dual nuclease activities: site‐specific (“cis”) cleavage of the target sequence and nonspecific (“trans”) collateral cleavage of single‐stranded DNA reporters [4, 5, 6]. This unique collateral activity serves as a novel specific signal–output tool, often coupled with nucleic acid amplification techniques in detection assays [7, 8]; however, in both in vitro diagnostics and in vivo genome editing applications, uncontrolled activation of Cas12a can lead to background signal and off‐target effects (the editing of DNA in unintended cells) [9, 10, 11], and a reliance on upstream amplification steps that complicate assay workflows and limit temporal or spatial precision [3, 12, 13].

Recently, efforts to regulate Cas12a activity have explored protein engineering [14, 15], chemical “caging” of guide RNAs [16, 17, 18], and logic‐gated circuit designs [19, 20, 21], yet these strategies often require elaborate synthesis, reduce reaction kinetics, or remain dependent on target preamplification to achieve clinically relevant sensitivity [22, 23, 24, 25]. In molecular diagnostics, isothermal amplification methods (e.g., Recombinase polymerase amplification, Loop‐mediated isothermal amplification) mitigate instrument requirements but can lead to primer‐induced amplification perturbations and extend assay times [26, 27, 28]. Meanwhile, amplification‐free CRISPR molecular diagnostics have advanced rapidly—spanning Cas12/13/14 collateral readouts, switchable/caged gRNA designs, and nucleic‐acid‐circuit‐driven cascade amplification [13, 23, 29, 30, 31, 32, 33, 34, 35]. Nevertheless, their broader deployment remains constrained—to varying degrees—by sensitivity limits, specificity, functional narrowness, and system complexity. In gene editing, spatiotemporal control over nuclease activation is critical to minimize unintended DNA damage and to enable conditional or programmable interventions in living cells or organisms [6, 10, 36]. Thus, a unified platform that affords both preamplification‐free detection and precise, target‐dependent editing in a single streamlined format remains an unmet need.

To address these challenges, we developed TopCas (Topology‐gated Cas12a via DNA‐RNA Chimeric Circular crRNA), building on the pivotal role of the crRNA in governing both target specificity and nuclease activation of Cas12a, as well as insights from RNA topology, Cas12a structural studies, and its trans‐cleavage activation characteristics [37, 38, 39, 40], together with the demonstrated utility of DNA–RNA chimeric nucleic acids in enhancing genome‐editing specificity and enabling sensitive nucleic‐acid diagnostics [33, 41, 42, 43]. Within the TopCas system, we engineered a DNA‐RNA chimeric circular crRNA that sterically constrains Cas12a's active conformation until linearization is triggered by target molecules. Upon target recognition, the assembled target‐crRNA‐Cas12a complex trans‐cleaves the DNA segment of the circular guide, converting it into its active linear form and initiating an autocatalytic activation cascade. This cascade obviates the need for external amplification steps and enables a one‐tube, one‐step fluorescent assay with high specificity and sensitivity. Moreover, the target‐activated TopCas system can also perform conditional gene editing: by incorporating a chimeric circular crRNA specific to an editing locus, the autocatalytic linear crRNA‐Cas12a complex acquires targeted cleavage activity, thereby enabling controlled gene editing with markedly reduced off‐target effects.

Subsequently, we elucidated the development and validation of the TopCas system. First, we achieved efficient intramolecular circularization of the chimeric crRNA, confirmed its capacity to constrain Cas12a activity, and investigated the factors modulating this inhibition. We then characterized the activation kinetics, specificity, and limit of detection using synthetic nucleic acid standards. Furthermore, we demonstrated TopCas's ability to accurately detect clinical specimens without preamplification and to monitor target molecules in live cells. Finally, we illustrated the system's versatility by performing conditional genome editing in mammalian cells, in which Cas12a activity is conditionally unleashed only in the presence of defined viral DNA or RNA targets—enabling precise editing of cellular genes. Collectively, these findings extend the capabilities of CRISPR‐Cas12a technology and lay a foundation for transformative clinical applications and next‐generation biotechnological innovations.

## 2 Results and Discussion

### 2.1 Design Principles of the TopCas Platform

The TopCas platform is built upon structural and functional insights into the substrate‐crRNA‐Cas12a ribonucleoprotein (RNP) complex. Formation of a catalytically active RNP requires proper and stable assembly of Cas12a with its guide RNA and target substrate. Concurrently, DNA and RNA can adopt higher‐order topologies when their termini are constrained [44, 45]. By engineering a circular crRNA to impose such topological constraints, we bias the RNP assembly toward catalytically incompetent states, thereby attenuating nuclease activity. Subsequent topological remodeling—achieved by linearization of the circular crRNA—restores the correct RNP conformation and reactivates Cas12a. Central to this mechanism is Cas12a's unique trans‐cleavage specificity for single‐stranded DNA and the design of the DNA‐RNA chimeric circular crRNA (CcrRNA).

**FIGURE 1:**
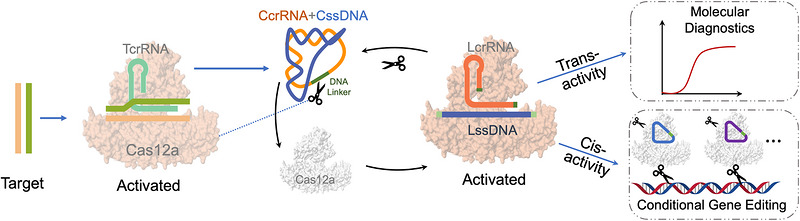
The schematic illustration of the chimeric circular crRNA‐Mediated CRISPR‐Cas12a platform (TopCas). The TcrRNA (Target‐activating linear crRNA)‐Cas12a complex recognizes and binds to the target nucleic acid, resulting in activation. The activated trans‐cleavage activity cleaves the DNA segment within the CcrRNA, thereby altering the topology between the CcrRNA and circular single‐stranded DNA (CssDNA) substrate and activating Cas12a nuclease activity. This activation triggers an autocatalytic cleavage cascade of the chimeric crRNA DNA region. Ultimately, the activated complex's trans‐cleavage activity facilitates signal amplification, while its cis‐cleavage activity enables precise gene editing.

In detail, a target nucleic acid molecule binds to a conventional linear crRNA‐Cas12a complex, activating the trans‐cleavage activity of the complex. This active RNP then selectively cleaves the single‐stranded DNA segment of the chimeric circular crRNA. Linearization of the circular guide converts it into an active crRNA‐Cas12a complex, which in turn cleaves additional circular crRNAs in an autocatalytic cascade. The fully activated Cas12a complex performs two parallel functions: trans‐cleavage of reporter molecules for fluorescent signal amplification, and cis‐cleavage of target DNA for precise gene editing (Figure 1).

### 2.2 Accurate Preparation of DNA–RNA Chimeric Circular crRNA

**FIGURE 2:**
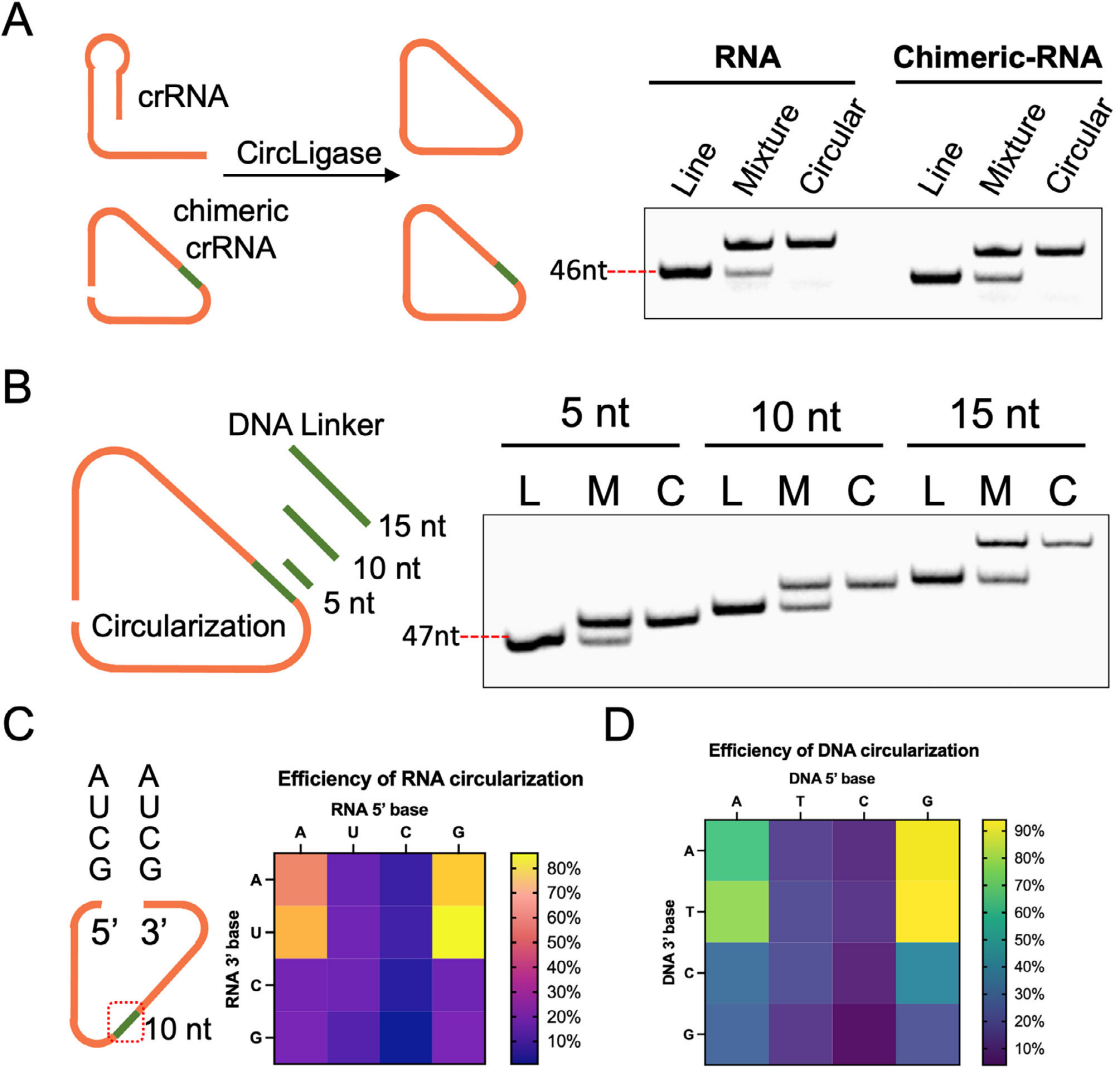
Preparation of circular nucleic acids using CircLigase. (A) Circularization of conventional crRNA and DNA‐RNA chimeric crRNA. (B) Circularization of crRNAs with different DNA segment lengths. L: linear; M: reaction mixture; C: circular. (C) Circularization efficiency of chimeric linear DNA‐RNA substrates with different 5′‐3′ nucleotide combinations (DNA segment length: 10 nt). (D) Circularization efficiency of ssDNA substrates with different 5′‐3′ nucleotide combinations. Data are means of three independent experiments (*n* = 3).

Formation of a precisely configured circular crRNA is essential to impose the intended topological constraint. Structural studies of the Cas12a‐DNA‐crRNA ternary complex (which revealed a fixed spatial separation between the crRNA's 5' and 3' termini in the active conformation; Figure S1) provided the structural basis for the rational design of a DNA fragment (in chimeric circular crRNA) with an appropriate length [37, 40]—namely, one that maximizes the topological suppression of Cas12a activity while remaining readily cleavable by the target‐activated trans nuclease activity of Cas12a. Conventional intramolecular cyclization of RNA or DNA typically employs T4 RNA ligase or DNA ligase in conjunction with a splint oligonucleotide; however, these methods often yield multimeric by‐products and oversized circles that are unsuitable for our platform. To overcome this, we employed a ligase with stringent intramolecular activity (CircLigase) [46] to generate monomeric circular crRNA exclusively (Figure S2). For the DNA‐RNA chimera, a chemically synthesized linear substrate bearing the desired RNA guide sequence flanked by DNA linkers was subjected to CircLigase‐mediated ligation, followed by sequential digestion with RNase R and Exonuclease I to remove any residual linear or concatenated species (Figure 2A). We further tested cyclization efficiency for chimeric crRNAs containing DNA linkers of various lengths (Figure 2B).

Next, we evaluated CircLigase's base‐pair preferences at the junction of 5′ and 3′ termini within the DNA‐RNA hybrid substrate, revealing significant sequence‐dependent differences in cyclization yield (Figure 2C). Kinetic analysis of the preferred 5′‐3′ base pairs was performed to establish the time course for complete circularization (Figure S3), and the exonuclease resistance of the resulting circular RNAs was confirmed under RNase R degradation (Figure S4). In addition, we investigated the cyclization efficiency of the circular substrate DNA that forms the topologically constrained architecture (Figure 2D). These optimization studies provided critical parameters for the high‐purity production of circular crRNA and underpinned the robustness of the TopCas platform by ensuring consistent guide topology and stability.

### 2.3 Topology‐Mediated Control of Cas12a Trans‐Cleavage by Chimeric Circular crRNA

**FIGURE 3:**
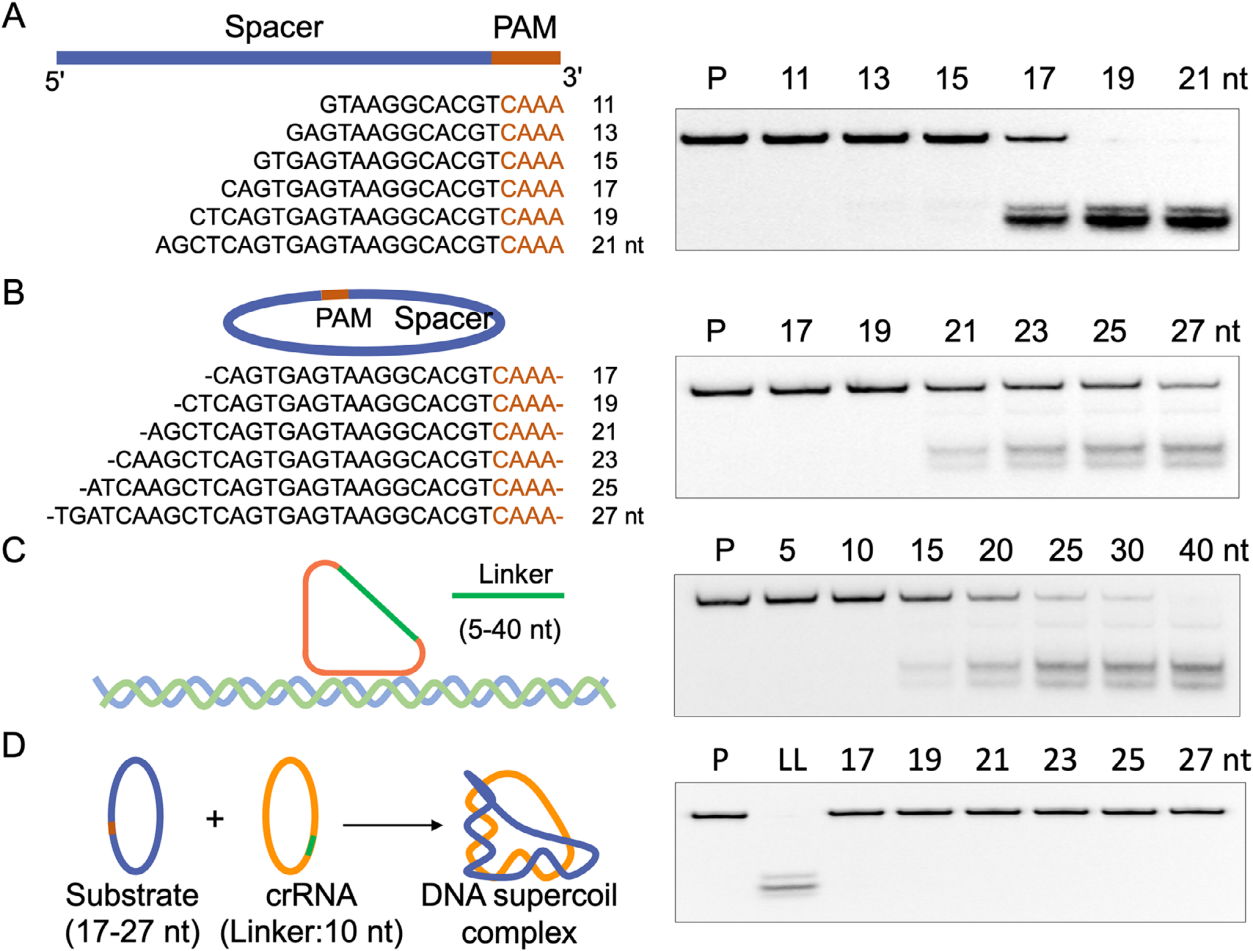
Comparison of Cas12a trans‐cleavage activation by different DNA substrates and crRNAs. (A) Activation of Cas12a trans‐cleavage activity by linear single‐stranded DNA substrates of varying lengths combined with linear crRNA; sequence information and denaturing urea‐PAGE analysis are shown. (B) Activation of Cas12a trans‐cleavage activity by circular single‐stranded DNA substrates of varying lengths combined with linear crRNA. (C) Activation of Cas12a trans‐cleavage activity by chimeric circular crRNAs with different DNA segment lengths in the presence of 200 bp double‐stranded DNA substrates. (D) Activation of Cas12a trans‐cleavage activity by circular single‐stranded DNA substrates of different lengths combined with chimeric circular crRNAs. P: the ssDNA probe as a blank control; LL: linear DNA substrates and linear crRNA, as a positive control. The experiments were conducted in three technical replicates.

Nucleic acids typically adopt higher‐order structures through base‐pair complementarity, yet a solitary circular crRNA may impose only limited topological constraints on Cas12a activity. To investigate this, we first assessed Cas12a trans‐cleavage activity against linear DNA substrates of varying lengths in the presence of circular crRNA (Figure S5). We observed that increasing substrate length progressively diminished trans‐cleavage rates, likely reflecting the formation of more complex topologies between longer DNA and the circular guide. This finding provided supporting evidence for the successful implementation of conditional gene editing with TopCas. Although long substrates paired with circular crRNA effectively suppressed Cas12a activity, ubiquitous exonucleases in biological milieus can truncate linear substrates, raising the risk of nonspecific Cas12a activation. Consequently, we evaluated circular DNA substrates capable of both resisting exonuclease digestion and imposing topological barriers. We determined the minimal lengths of single‐strand linear and circular DNA required for Cas12a activation, and examined duplex substrates paired with circular crRNAs of varying sizes (Figure 3A–C). Our determinations of the minimal activating lengths for linear and circular substrates are consistent with—and further substantiate—observations reported by Rananaware, S. R., [47]and by Deng. F. et al. [20]. Notably, a narrow topological “window” of 4 nt emerged, wherein single‐strand linear spacer≥15 nt and circular spacer ≥19 nt optimally modulated trans‐cleavage. This limits the scope for creating switches that function through substrate topological variation. In addition, linear substrates still exhibited suboptimal control when used alone across different circular crRNA sizes.

To enhance this topological control capability, we next interrogated the combined effect of circular substrates and circular crRNA on Cas12a trans‐cleavage (Figure 3D). Robust suppression was observed across all tested substrate sizes, effectively widening the topology circular‐to‐linear transition window. To further verify the effect of circular substrates and circular crRNA formation topology on Cas12a activity, we performed a dot blot experiment (Figure S6). The results showed that compared with linear crRNA, circular crRNA formed less complexes with Cas12a protein, and the circular substrate further weakened the binding ability of the circular crRNA‐Cas protein complex. Compared with the larger circular crRNA alone [48], the complex formed by the circular substrate and the circular crRNA exhibited an even more pronounced reduction in binding affinity toward Cas12a. To directly link these observations to higher‐order topology, we engineered circular single‐strand DNA to form tetrahedral architectures (Figure S7A). Linear DNA progressively assembled into multimeric polymers as component concentration increased (Figure S7B), while circular DNA impeded tertiary complementation after dimer formation (Figure S7C). Upon addition of linear substrates, this inhibitory effect was alleviated, allowing polymerization to proceed (Figure S7D). In addition, native gel electrophoresis (non‐denaturing PAGE) and melting curve analysis revealed the formation of higher‐order assemblies between linear/circular crRNA and ssDNA substrates (Figure S7E,F). These findings indicated that two complementary circular oligonucleotides likely generate complex topologies—key to the observed suppression and activation of Cas12a trans‐cleavage by our chimeric circular crRNA system.

### 2.4 Topology‐Specific Modulation Establishes the TopCas Platform

**FIGURE 4:**
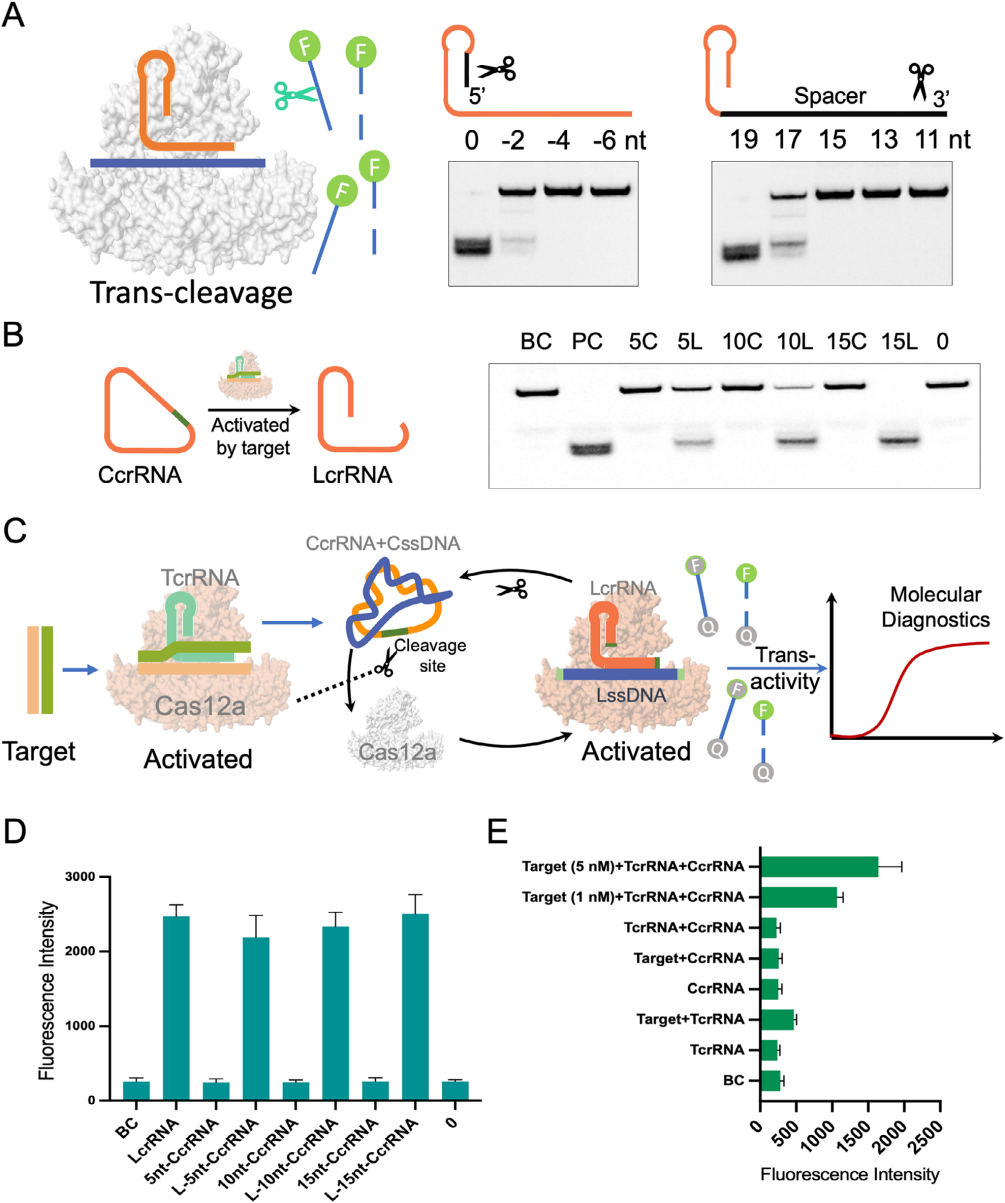
TopCas molecular diagnostic platform mediated by chimeric circular crRNA and activated via topology changes to induce Cas12a trans‐cleavage activity. (A) Determination of the shortest crRNA capable of activating Cas12a trans‐cleavage activity. crRNAs were sequentially truncated from the 5′ and 3′ ends, and Cas12a activity was assessed by the degradation of fluorescent reporter molecules analyzed via denaturing urea‐PAGE. (B) Verification that DNA‐RNA chimeric circular crRNAs (CcrRNAs) containing DNA segments of varying lengths (5–15 nt) can be linearized by activated Cas12a‐crRNA‐DNA complexes and subsequently reused in the Cas12a system. BC: blank control; PC: positive control (linear crRNA‐DNA activates Cas12a trans‐cleavage); 0: no second Cas12a addition group. (C) Schematic illustration of the TopCas molecular diagnostic platform. TcrRNA: target‐specific crRNA; CcrRNA: chimeric circular crRNA; LcrRNA: linearized form of CcrRNA; CssDNA: circular single‐stranded DNA substrate; LssDNA: linearized form of CssDNA. (D) Validation of fluorescence reporter cleavage (FAM‐BHQ1) corresponding to the experiment in (B). (E) Fluorescence signal analysis of TopCas system components; BC: blank control containing all components except crRNA. For (D) and (E), the experiments were conducted in three technical replicates, and error bars represent mean ± SD (*n* = 3).

Precise control of Cas12a activation via modulation of crRNA‐circDNA topology is fundamental to the TopCas design. Activated Cas12a indiscriminately cleaves single‐stranded DNA substrates while exhibiting no activity toward RNA, rendering reliance on trans‐cleavage of a circular substrate inherently stochastic. To overcome this, we paired a nuclease‐resistant, stably circularized DNA substrate with a tunable circular crRNA to achieve both precise and reproducible activation. Substrate stability was enhanced through phosphorothioate modifications introduced at various nucleotide segments and positions [49, 50], and the impact of these modifications on Cas12a trans‐cleavage kinetics was systematically evaluated (Figure S8). Linearization of the circular crRNA was affected by specific trans‐cleavage of its DNA segment within the DNA‐RNA chimera. We then mapped the sequence‐dependent preferences of activated Cas12a for substrate cleavage (Figure S9A), performed kinetic analyses of collateral cleavage on chimeric substrates (Figure S9B), and performed structural modeling of the chimeric crRNA (Figure S10), revealing that certain junctional sequences permit efficient cleavage without imposing additional higher‐order folding on the guide—thereby facilitating rapid ring‐opening. Recognizing that larger RNA constructs are prone to nonspecific fragmentation, we determined the minimal linear crRNA length required to sustain Cas12a activation (Figure 4A), observing a progressive decline in trans‐cleavage activity as the 5′ and 3′ termini were truncated. Moreover, we assessed activation by circular crRNAs bearing defined nicks at various breakpoints (Figure S11) and by randomly cleaved circles (Figure S12), demonstrating that site‐specific chemical modifications can suppress undesirable background activation. Collectively, these optimization studies provide essential design parameters for generating stable, responsive chimeric crRNAs.

We then validated the TopCas mechanism in vitro. Upon target‐triggered activation, Cas12a trans‐cleaves the circular crRNA, converting it into a linear guide and initiating a self‐amplifying cascade of trans‐cleavage events. Chimeric guides with varied DNA linker lengths were all efficiently linearized and activated Cas12a trans‐cleavage, as confirmed by direct enzyme assays (Figure 4B) and fluorogenic reporter release (Figure 4D). Specific activation by DNA targets yielded robust fluorescence signals only in target‐positive samples, scaling with target concentration and remaining minimal in negative controls (Figure 4E). Although the endpoint fluorescence values differed markedly between the autocatalytic activation group (Target + TcrRNA + CcrRNA) and the non‐autocatalytic group (Target + TcrRNA), the signal‐to‐noise ratio remained suboptimal, likely owing to the relatively high target concentration and a not‐yet fully optimized reaction configuration. Extending our investigation to RNA triggers—given literature precedence for RNA‐mediated Cas12a activation [19, 51, 52]—we observed that RNA targets can also induce trans‐cleavage, albeit less efficiently than DNA at equivalent concentrations (Figures S13 and S14). These findings collectively establish a topology‐gated, target‐specific activation framework that underlies the TopCas platform.

### 2.5 Systematic Optimization of the TopCas Platform

**FIGURE 5:**
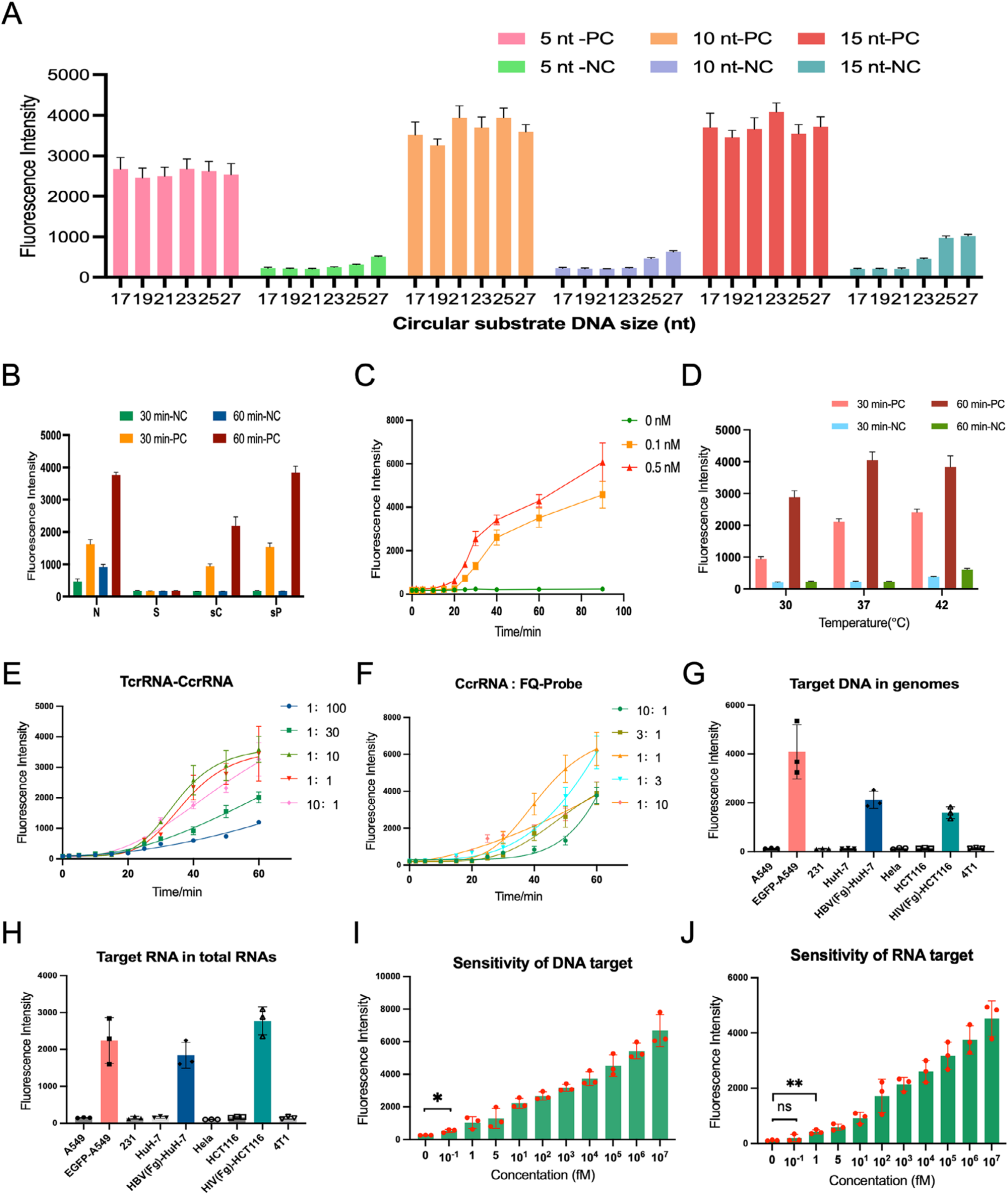
Optimization, Specificity, and Sensitivity of the TopCas system. (A) Combinations of CcrRNAs of varying lengths with circular DNA substrates (spacer) of different sizes in the TopCas system. (B) Signal output of TopCas systems containing various chemically modified circular DNA substrates in nuclease resistance assays (0.1% fetal bovine serum was added to the standard TopCas reaction mixture), N: native circular DNA, S: the phosphorothioate‐modified circular ssDNA, sC: the circular ssDNA with cleavage site modified by phosphorothioate, sP: the circular ssDNA with PAM modified by phosphorothioate. (C) Time‐course fluorescence profiles of the TopCas system containing 10 nt CcrRNA and 21 nt circular ssDNA with phosphorothioate modification at the PAM site at different target concentrations. (D) Fluorescence signal output of the TopCas system at different reaction temperatures (0.5 nm target, 10 nt CcrRNA, and 21 nt PAM‐PS‐modified circular ssDNA). (E) Signal output with varying ratios of target crRNA (TcrRNA) to chimeric circular crRNA (CcrRNA). (F) Signal output with varying ratios of CcrRNA to FAM‐Quencher (FQ) probe molecules (other TopCas components and reaction conditions were as in (D) at 37°C). (G) Target DNA is detected in complex nucleic acid samples using the TopCas system, the target including EGFP, HBV(Fg), HIV(Fg), and the genome as background DNA. (H) Target RNA is detected in complex nucleic acid samples using the TopCas system, the target including EGFP, HBV(Fg), HIV(Fg), and the total genome RNA as background RNA. (I) The sensitivity of the TopCas system in detecting DNA. (J) The sensitivity of the TopCas system in detecting RNA. The experiments were conducted in three technical replicates, and error bars represent mean ± SD (*n* = 3), Statistical differences between groups were determined using one‐way ANOVA with Tukey post‐tests. *p* < 0.05 (*); *p* < 0.01 (**); ns: not significant.

To refine the TopCas system, we performed a series of targeted optimizations. First, the length of the DNA linker within the chimeric circular crRNA (CcrRNA) was identified as a key determinant of both topological constraint and enzyme‐activation kinetics. We therefore compared CcrRNAs bearing 5‐nt, 10‐nt, and 15‐nt linkers (Figure 5A). The 5‐nt CcrRNA produced weaker fluorescence and required longer incubation to reach maximal signal, whereas the 15‐nt CcrRNA yielded elevated background fluorescence in negative controls. In contrast, the 10‐nt CcrRNA balanced rapid signal generation with low background, demonstrating superior signal‐to‐noise performance. Further, we performed region‐specific phosphorothioate modifications on the circular DNA substrate to enhance its biological stability, thereby improving the accuracy of the TopCas system (Figure 5B). Using a circular DNA substrate with phosphorothioate modifications in the PAM (PAM‐PS) region, we performed a time‐course analysis of the 10‐nt CcrRNA, which thereby confirmed its optimal kinetic profile (Figure 5C). Additionally, we investigated the reaction temperature for the TopCas system. The results showed that higher temperatures correlated with increased fluorescence signal intensity, but also produced a concomitant increase in the background signal of the negative controls (Figure 5D).

Next, we optimized the ratio of target‐activating linear crRNA (TcrRNA) to circular crRNA (CcrRNA). Insufficient TcrRNA reduced RNP assembly and delayed activation, whereas excess TcrRNA sequestered Cas12a into inactive complexes, attenuating the autocatalytic amplification and diminishing fluorescence output. A TcrRNA:CcrRNA ratio between 1:10 and 1:30 provided the most efficient signal generation (Figure 5E). Similarly, the balance between CcrRNA and reporter substrate was critical: a 1:1 ratio maximized autocatalytic turnover without degrading the reporter pool (Figure 5F). We also compared simple fluorogenic reporters with CcrRNA constructs bearing built‐in signal‐release motifs (Figure S15). Both formats yielded comparable signals; however, chemically synthesized signal‐integrated CcrRNAs incurred substantially higher cost, making conventional reporters the more economical choice in most applications. Collectively, these optimization efforts culminated in a robust TopCas configuration—employing a 21 nt PAM‐PS circular ssDNA substrate, a 10‐nt DNA linker CcrRNA, a 1:10–1:30 TcrRNA:CcrRNA ratio, and a 1:1 CcrRNA:reporter ratio—that delivers reliable, high‐contrast fluorescence and efficient, reproducible activation across molecular detection and editing workflows.

### 2.6 Specificity and Sensitivity of Molecular Detection by the TopCas System

The specificity and sensitivity of molecular detection are critical benchmarks for evaluating the clinical utility of the TopCas platform. To assess specificity, we first chemically synthesized standard target nucleic acid sequences and performed cross‐detection assays using the TopCas system. The results demonstrated that the TopCas system exhibited high specificity in detecting the standard targets (Figure S16). Then, we cloned target sequences (or fragments thereof) into plasmids and transfected them into various cell lines. Genomic DNA and total RNA were extracted and analyzed by qPCR and RT‐qPCR (Figure S17), respectively, as the gold‐standard reference assays. Parallel samples were subjected to detection on the TopCas system and the results compared. As shown in Figure 5G,H, TopCas accurately discriminated target nucleic acids even within complex background samples, displaying specificity equivalent to that of PCR‐based methods.

To evaluate sensitivity, we performed serial dilutions of target DNA and RNA and measured the detection limits of TopCas. The platform reliably detected target DNA at concentrations as low as 100 aM, whereas RNA targets required higher input levels for robust activation (Figure 5I,J), consistent with the differential activation efficiency of Cas12a by RNA. Our TopCas system enables low‐abundance quantification of DNA and/or RNA targets, delivering sensitivity that is comparable to—or, in certain contexts, exceeds—recently reported amplification‐free CRISPR assays [31, 32, 33, 53], while providing distinctive advantages in assay time and operational simplicity. Notably, TopCas achieves these performance metrics without any preamplification steps, thereby greatly simplifying assay composition and enhancing both temporal and spatial efficiency.

### 2.7 Clinical Sample and Intracellular Target Detection Using the TopCas Molecular Diagnostic Platform

**FIGURE 6:**
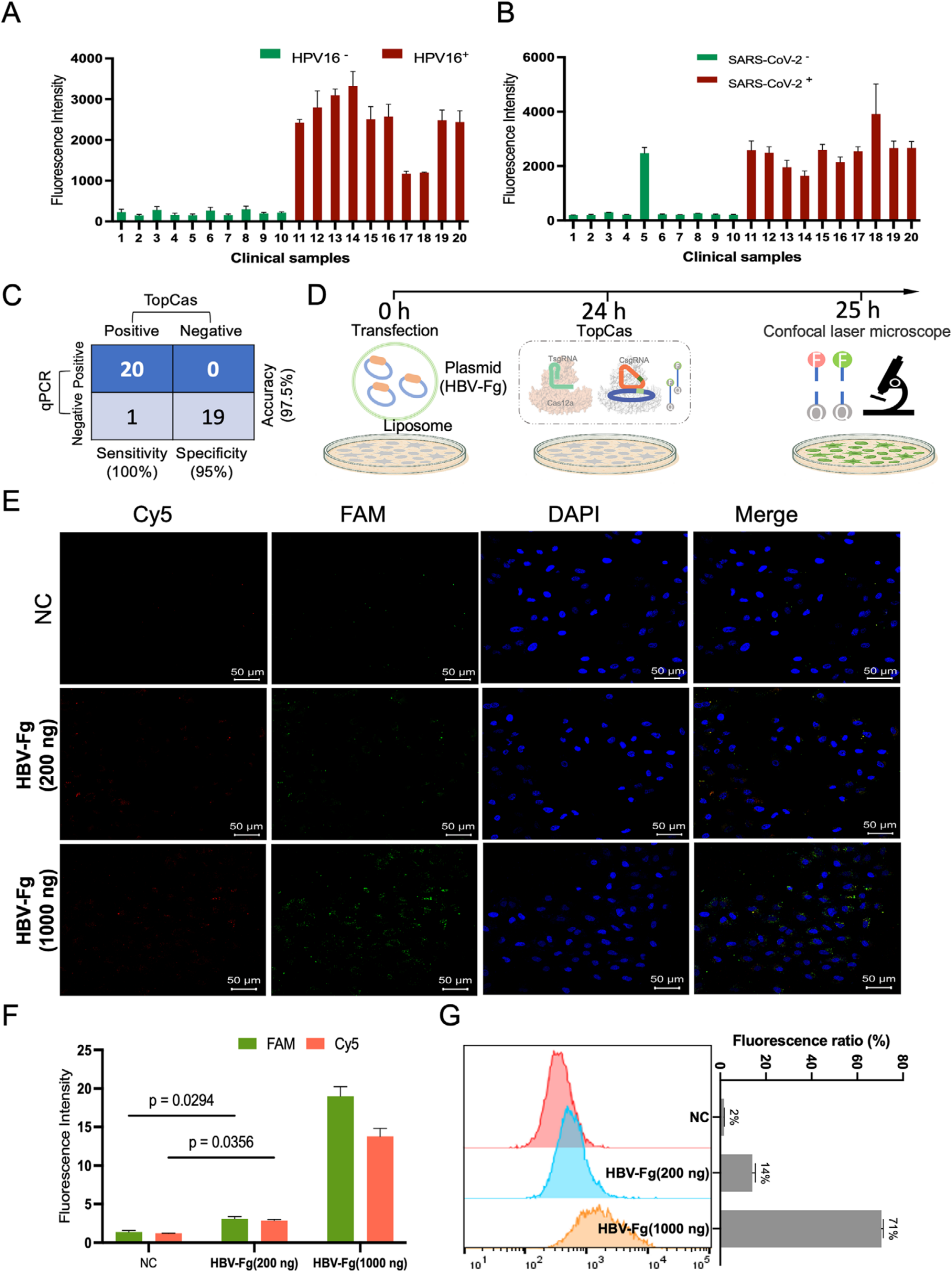
Clinical sample detection and intracellular target molecule analysis using the TopCas system. (A) Detection of HPV16 L1 DNA in qPCR‐confirmed HPV16‐positive and negative clinical nucleic acid samples by TopCas. (B) Detection of SARS‐CoV‐2 N gene RNA in qPCR‐confirmed positive and negative clinical samples using TopCas. Experiments were performed in triplicate; error bars represent mean ± SD (*n* = 3). (C) Sensitivity and specificity assessment of the TopCas molecular diagnostic platform compared to qPCR. (D) Schematic illustration of intracellular target molecule detection. (E) Target molecules were transfected into cells, and fluorescent signal release by the TopCas system was detected by laser scanning confocal microscopy. NC: negative control without HBV‐Fg plasmid; Cy5 and FAM represent a dual‐fluorescence reporter system. Scale bar: 50 µm. (F) Quantitative analysis of Cy5 and FAM fluorescence signals from (E), statistical differences between groups were determined using one‐way ANOVA with Tukey post‐tests. (G) Flow cytometry analysis of fluorescence and quantification of fluorescence‐positive cell populations from (E). Data are presented as mean ± SD (*n* = 3).

To evaluate the clinical diagnostic performance of the TopCas system, we collected DNA from qPCR‐confirmed HPV16‐positive and HPV16‐negative clinical specimens, as well as RNA from SARS‐CoV‐2‐positive and ‐negative patient samples (Table S4). These nucleic acid extracts were assayed on the TopCas platform, and fluorescence outputs were compared to reference qPCR/RT‐qPCR results. For DNA targets, both positive and negative predictive agreements with qPCR reached 100% (Figure 6A). For RNA targets, the positive predictive agreement was 100% and the negative predictive agreement 95% (Figure 6B,C). These metrics match or exceed those reported for other Cas12a‐based detection systems, underscoring TopCas's robust clinical performance.

Taking advantage of the isothermal and room‐temperature characteristics of the detection system, we further explored its capacity for intracellular target detection. HuH‐7 cells were lipofected with plasmids encoding hepatitis B virus (HBV) genome fragments, and intracellular nucleic acids were probed directly by TopCas to generate fluorescence signals. Transfected cells exhibited strong, target‐dependent fluorescence that correlated with plasmid dose (Figure 6D,E). Flow cytometric analysis confirmed these findings (Figure 6F). The results demonstrated that the TopCas system successfully identified intracellular target molecules and initiated signal release, with the signal intensity showing a positive correlation with target concentration. Collectively, these results demonstrated TopCas's versatility for both clinical sample diagnostics and live‐cell molecular imaging, highlighting its potential for diverse in vitro and in vivo applications.

### 2.8 Conditional Gene Editing Enabled by the TopCas Platform

**FIGURE 7:**
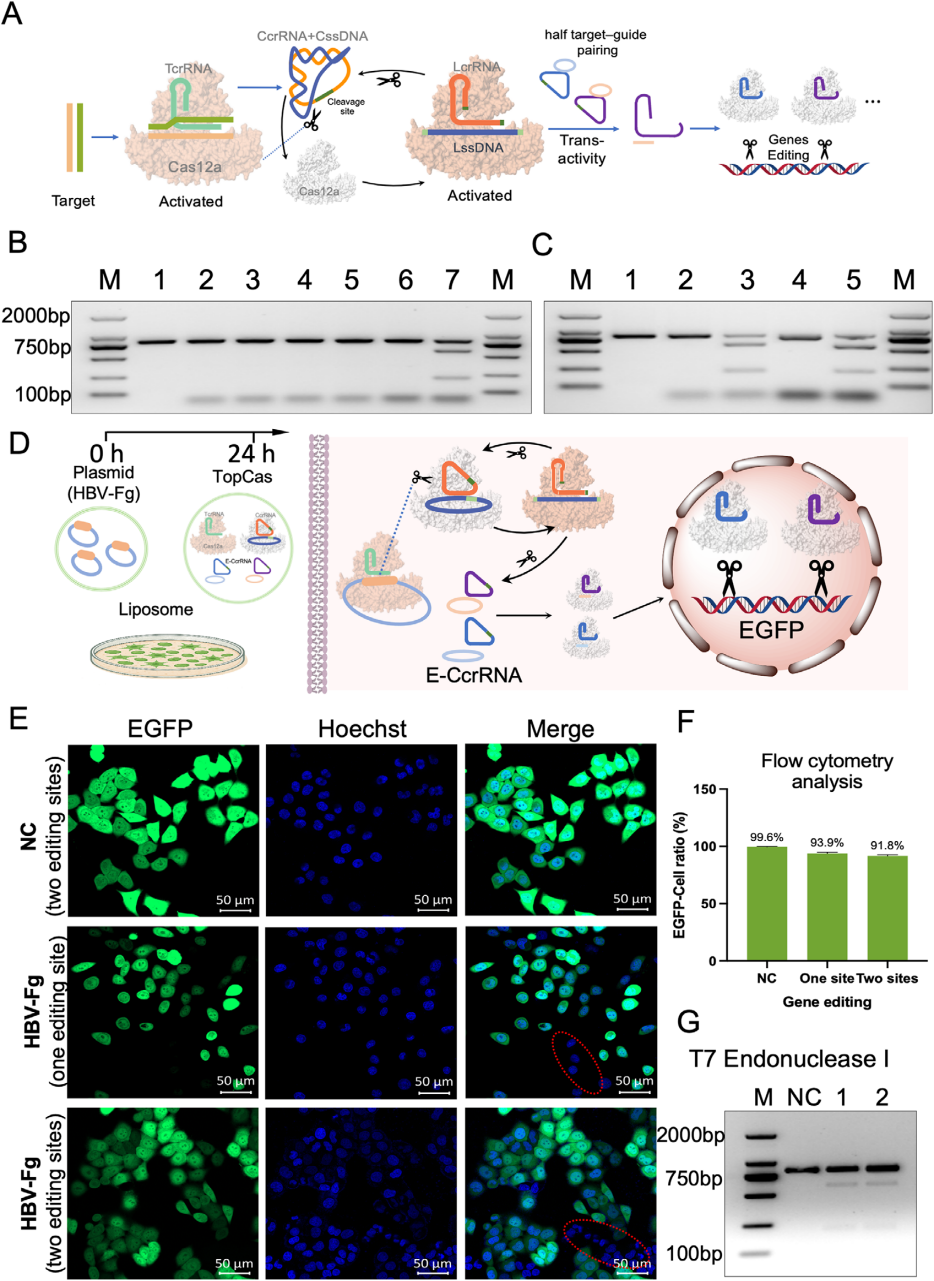
Conditional gene editing using the TopCas system. (A) Schematic overview of conditional gene editing mediated by the TopCas system. (B) Cis‐cleavage activity of the TopCas system on target DNA with various component combinations. M: DNA marker; 1: target edited DNA; 2: TcrRNA only; 3: trigger DNA + TcrRNA; 4: CcrRNA + CssDNA; 5: trigger DNA + CcrRNA + CssDNA; 6: TcrRNA + CcrRNA + CssDNA; 7: trigger DNA + TcrRNA + CcrRNA + CssDNA. All reactions (1‐7) contained Cas12a protein and standard reaction buffer. (C) Multi‐target gene cleavage activity. 1: target edited DNA; 2: single‐target cleavage system without trigger DNA; 3: single‐target cleavage system with trigger DNA; 4: dual‐target cleavage system without trigger DNA; 5: dual‐target cleavage system with trigger DNA. (D) Schematic illustration of intracellular conditional gene editing by the TopCas system with HBV‐Fg as the trigger target. (E) Laser confocal microscopy analysis of EGFP fluorescence in cells subjected to TopCas‐mediated gene editing. “One editing site” indicates one editing site in the EGFP gene; “two editing sites” indicates two editing sites in the EGFP gene. Scale bar: 50 µm. Red dashed circles indicate cells in which the EGFP gene has been edited. (F) Flow cytometry quantification of fluorescent cells corresponding to (E). (G) T7 Endonuclease I assay (New England Biolabs) analyzing target gene editing in cells from (E). M: DNA marker; NC: without HBV‐Fg plasmid; 1: one kind of E‐CcrRNA for one editing site; 2: two kinds of E‐CcrRNA fort wo editing sites; Data are presented as mean ± SD (*n* = 3).

Uncontrolled nuclease activity poses substantial risks to normal cellular functions, thereby constraining the therapeutic applicability of gene editing. To surmount this challenge, we leveraged the inherent conditionality of the TopCas platform to achieve target‐dependent activation of Cas12a‐mediated editing. In this configuration, the presence of specific intracellular nucleic acids (e.g., HBV or HIV) initiates the same autocatalytic cascade underlying TopCas diagnostics; however, the fluorescent reporter is supplanted by a chimeric circular crRNA designed to target the genomic locus of interest. Beyond the diagnostic topology (CcrRNA–CssDNA) that becomes activatable upon linearization to drive the autocatalytic loop, the gene‐editing module employs a “half‐paired” design in which only 11 nt of the spacer anneal to the crRNA (It was shown in Figure 3A that Cas12a could not be activated), thereby substituting for the reporter. Even after linearization, this editing CcrRNA (E‐CcrRNA) cannot activate Cas12a. This design both preserves the topological suppression that prevents nonspecific Cas12a activation and avoids depletion of cis‐cleavage capacity by trans‐cleavage–driven activation. Target‐triggered trans‐cleavage of the crRNA's DNA moiety linearizes the guide, which then assembles with Cas12a to form an active ribonucleoprotein complex capable of site‐specific (“cis”) cleavage (Figure 7A).

Mechanistic validation in vitro confirmed that cleavage of a model DNA substrate occurred exclusively under conditions comprising both the cognate trigger molecule and a complete TopCas reagent set (Figure 7B; Figure S18). Further, by incorporating multiple distinct chimeric crRNAs within a single reaction, we would demonstrate multiplexed, tunable cleavage of several genomic targets (Figure 7C). The results demonstrated that only the complete TopCas components enabled target DNA cleavage, while also supporting conditional cleavage at multiple loci, thereby showcasing the system's stringent conditional activation capability and multiplex gene editing potential. In cellular assays, EGFP‐expressing A549 cells were co‐transfected with plasmids encoding HBV genome fragments, followed by lipofection of TopCas editing mixtures containing an EGFP‐targeting crRNA alongside either HBV‐specific triggers (Figure 7D; Figure S19). EGFP expression was analyzed by confocal microscopy and flow cytometry (7E‐F). The results demonstrated that groups with the HBV fragment plasmid, which triggers gene editing, exhibited reduced or absent target gene expression. Further quantitative analysis revealed a statistically significant decrease in the proportion of EGFP‐positive cells in the HBV‐triggered group compared to the control group lacking the HBV fragment‐encoding plasmid. On‐target genomic modifications were further substantiated using a mutation detection assay (Figure 7G), thereby confirming the occurrence of conditional gene editing events in live cells. In this study, HBV‐triggered editing was qualitatively evaluated by confocal imaging and T7 endonuclease I analysis and coarsely quantified by flow cytometry, with the primary objective of establishing a conditional gene‐editing model. We note that the observed editing efficiencies are lower than those typically reported for conventional Cas12a or Cas9 systems [15, 54, 55, 56, 57], which is plausibly attributable to the additional gating step intrinsic to TopCas—namely, activation strictly contingent on the presence of the target nucleic acid. Collectively, these findings establish TopCas as a versatile, programmable platform that enables conditional and multiplex gene editing, offering a promising model for precision therapeutics and disease prevention.

## 3 Conclusion

In this work, we have developed and thoroughly validated the TopCas platform, which harnesses the unique topology‐gated activation of Cas12a via DNA‐RNA chimeric circular crRNAs. The ability to precisely and conditionally activate nuclease activity is critical for both reducing off‐target effects in gene editing and minimizing background noise in molecular diagnostics. This innovative strategy enables precise spatial and temporal control of Cas12a nuclease activity, effectively overcoming key challenges associated with nonspecific activation and dependence on nucleic acid preamplification that commonly hinder current CRISPR‐based diagnostics and gene editing systems. Through systematic optimization of linker lengths, chemical modifications, and substrate design, TopCas achieves robust and reproducible activation with high specificity and sensitivity, comparable to PCR‐based workflows while obviating preamplification. The platform's versatility was demonstrated across a broad spectrum of applications, including highly accurate detection of clinically relevant pathogens such as HPV16 and SARS‐CoV‐2, as well as real‐time intracellular monitoring of viral nucleic acids. Importantly, TopCas also enables programmable gene editing conditioned on the presence of disease‐specific nucleic acid triggers. This feature was validated in vitro and in mammalian cell models, where precise and multiplexed genomic modifications were achieved with minimized off‐target effects. Such conditional editing holds significant potential for safer and more effective gene therapies, although there remains considerable room for improvement in editing efficiency—with potential contributing factors including delivery systems and the intrinsic cis‐cleavage activity of Cas12a [56, 58, 59]. Collectively, TopCas represents a unified, modular system that seamlessly integrates sensitive molecular diagnostics with controllable genome editing. Its topology‐based activation mechanism simplifies assay workflows, reduces background noise, and enhances target specificity, thereby advancing the frontiers of CRISPR biotechnology. Moving forward, further in vivo validation, delivery optimization, and expansion of the target scope will be critical to translate this promising technology into clinical and industrial applications.

## 4 Experimental Section

### 4.1 Synthesis of DNA‐RNA Chimeric Circular crRNA

DNA‐RNA chimeric linear oligonucleotides, chemically synthesized with a 5′‐phosphate and 3′‐hydroxyl group (Sangon Biotech Co., Ltd.), were circularized using CircLigase (HaiGene Biotech Co.,Ltd) according to the manufacturer's protocol with minor modifications. Briefly, 100 pmol of the linear oligonucleotide was incubated in a 20 µL reaction containing 8 µL of 2.5 × CircLigase Reaction Buffer, 1 µL of 50 mm MnCl_2_, 2 µL of CircLigase (200 U), and nuclease‐free water to volume. The mixture was thoroughly mixed and incubated at 55°C for 60 min. To terminate the reaction, the mixture was heat‐inactivated at 85°C for 10 min.

Next, un‐ligated linear oligonucleotides were degraded by treating the reaction with 2 µL of exonuclease III and RNase R in a total volume of 50 µL (containing 20 µL of the Cir‐DNA‐RNA product and 1×RNase R Reaction Buffer). The mixture was incubated at 37°C for 120 min and then subjected to extraction with an equal volume of phenol: chloroform: isoamyl alcohol (25:24:1, v/v/v) to remove proteins. The aqueous phase was recovered, and circular crRNA was precipitated by addition of 3 volumes of ice‐cold ethanol to a final concentration of 75% (v/v), followed by incubation at −20°C overnight. The sample was centrifuged at high speed (≥12,000 × g) for 20 min at 4°C, the supernatant was carefully removed, and the pellet was air‐dried at room temperature. The dried pellet was resuspended in nuclease‐free water containing RNase inhibitor (final concentration: 0.1 U/µL). The concentration of crRNA was determined, and aliquots were stored at −80°C in aliquots. All crRNA and DNA circularization reactions in this study were performed using the protocol described above, with the only variable being the sequence and length of the linear substrate oligonucleotide. For reactions employing T4 RNA ligase, all procedures were strictly followed the manufacturer's instructions (New England Biolabs) and are not described in detail herein.

### 4.2 Establishment of the TopCas Detection Platform

The TopCas detection platform is based on a topology‐controlled self‐catalytic system, utilizing circular substrates and chimeric circular crRNA. A standard 30 µL reaction mixture contained: 3 µL of 10× NEBuffer r2.1, 1 µL of 1 µm LbCas12a endonuclease, 1 µL of 0.5 µm target crRNA (TcrRNA), 30 nm (final concentration) chimeric circular crRNA (CcrRNA), 30 nm (final concentration) circular single‐stranded DNA (ssDNA) substrate, 1 µL of 10 µm FAM‐labeled ssDNA reporter (5′‐FAM‐ssDNA‐BHQ1), 0.5 U/µL RNase inhibitor, and nuclease‐free water to a final volume of 30 µL. An optimized 30 µL reaction mixture contained 3 µL of 10× NEBuffer r2.1, 1 µL of 1 µm LbCas12a endonuclease, 5 nm target crRNA (TcrRNA), 50 nM (final concentration) chimeric circular crRNA (CcrRNA), 50 nm (final concentration) circular single‐stranded DNA (ssDNA) substrate, 50 nm FAM‐labeled ssDNA reporter (5′‐FAM‐ssDNA‐BHQ1), 0.5 U/µL RNase inhibitor, and nuclease‐free water to a final volume of 30 µL.

The reaction mixture was incubated at 37°C for 15 min to allow the interaction between the circular crRNA and the circular substrate. Following this incubation, the remaining components were added to initiate the full reaction. Fluorescence intensity, reflecting the release of FAM‐labeled ssDNA, was measured to assess the activation of the TopCas system by different concentrations of target DNA or RNA molecules. To optimize the TopCas detection platform, individual components of the reaction mixture were systematically varied, with all other components held constant. This approach allowed for the identification of optimal concentrations and conditions for target detection and Cas12a activation.

### 4.3 Specificity and Sensitivity Testing of the TopCas System

The specificity and sensitivity of the TopCas detection platform were evaluated by assessing its performance across a range of target nucleic acids and under varying experimental conditions. Specificity Testing: Chemically synthesized DNA and RNA oligonucleotides, as well as nucleic acids extracted from cells, were used as substrates for specificity analysis. (1) Chemically synthesized sequences with distinct target and non‐target sequences were directly tested using the optimized TopCas reaction system; (2) For cellular nucleic acids, plasmids containing target fragments (EGFP, HBV‐Fg, and HIV‐Fg) cloned into pCMV (Kozak) vectors were transfected separately into A549, HuH‐7, and HCT116 cell lines using Lipofectamine 3000. After 48 h of culture, total DNA and RNA were extracted from cells using commercial kits (OMEGA) according to the manufacturer's instructions; (3) The TopCas reaction mixture was supplemented with 200 ng of total DNA or RNA (from either target‐containing or control groups), and endpoint fluorescence measurements were taken to quantify fluorescence intensity differences among the groups. qPCR detection of target nucleic acids in a complex background with the same nucleic acid samples. Sensitivity Testing: Sensitivity was assessed by serial dilution of target nucleic acid sequences in tenfold steps, starting from 10 nm down to 0.1 fM. The optimized 30 µL TopCas reaction system was used for each dilution point, and fluorescence intensity corresponding to target detection was recorded. The limit of detection (LOD) was defined as the lowest target concentration at which the fluorescence signal could be reliably distinguished from background noise with statistical significance.

### 4.4 Clinical Sample Testing Using the TopCas System

Clinical nucleic acid samples targeting DNA were obtained from 10 HPV16‐positive and 10 HPV16‐negative specimens, as confirmed by quantitative PCR (qPCR). RNA target samples comprised 10 SARS‐CoV‐2‐positive and 10 SARS‐CoV‐2‐negative specimens confirmed by reverse transcription qPCR (RT‐qPCR). All samples were obtained from the First Affiliated Hospital of Army Medical University. The study protocol was formally approved by the Ethics Committee of the First Affiliated Hospital of Army Medical University (Approval No.: KY2020270). For each assay, 5 µL of extracted nucleic acid was added to the optimized TopCas reaction mixture containing the corresponding target‐specific crRNA (TcrRNA). The reaction mixtures were incubated at 37°C for 1 h, after which fluorescence intensity was measured. Each clinical sample was tested in triplicate to ensure reproducibility. The diagnostic accuracy and sensitivity of the TopCas system were evaluated by comparing the assay results to the respective PCR‐based detection outcomes.

### 4.5 Intracellular Target Molecule Detection Using the TopCas System

HuH‐7 cells were seeded at a density of 5.0 × 10^5 cells per well and transfected with 0, 200, and 1000 ng plasmids encoding hepatitis B virus (HBV) genome fragments using Lipofectamine 3000 (Thermo Fisher Scientific) according to the manufacturer's instructions. Cells were incubated for 24 h post‐transfection to allow expression of the target nucleic acids. For intracellular detection, cells were fixed in cold fixative solution (methanol: acetic acid, 3:1, v/v) for 20 min at room temperature. Following fixation, cells were permeabilized with phosphate‐buffered saline (PBS) containing 0.5% (v/v) Triton X‐100 at 37°C for 5 min. Cells were then washed three times with 1× PBS at 37°C. Subsequently, cells were incubated with a 300 µL TopCas detection mixture containing 3 µL of 10× NEBuffer r2.1, 1 µL of 1 µm LbCas12a endonuclease, 5 nm HBV target crRNA (TcrRNA), 50 nm (final concentration) chimeric circular crRNA (CcrRNA), 50 nm (final concentration) circular single‐stranded DNA (ssDNA) substrate, 25 nm FAM‐labeled ssDNA reporter (5′‐FAM‐ssDNA‐BHQ1), 25 nm CY5‐labeled ssDNA reporter (5′‐CY5‐ssDNA‐BHQ3), 0.5 U/µL RNase inhibitor, and nuclease‐free water to a final volume of 300 µL at 37°C for 1 h. After incubation, cells were counterstained with 4′,6‐diamidino‐2‐phenylindole (DAPI) to visualize nuclei and subjected to confocal laser scanning microscopy (ZEISS LSM900) for fluorescence imaging of intracellular target molecules. For flow cytometry analysis, cells treated with the TopCas system as described above were harvested by trypsinization, washed three times with PBS, and maintained on ice in PBS. A total of 1 × 10^6 cells per sample were analyzed using a FACSymphony A1 equipped with appropriate lasers and filters to detect the FAM fluorescence indicative of Cas12a trans‐cleavage activity. Data were processed to quantify intracellular target detection efficiency and specificity.

### 4.6 In Vitro Validation of TopCas‐Mediated Gene Editing

The TopCas system was used to simulate gene editing in vitro. Target DNA substrates were generated by conventional PCR amplification of the EGFP gene segment using primers designed for the specific locus. PCR products were purified using a PCR purification kit (OMEGA) and analyzed by agarose gel electrophoresis to confirm size and purity. For the gene editing assay, 200 ng of purified PCR product was added to the TopCas reaction mixture consisting of 3 µL of 10× NEBuffer r2.1, 1 µL of 1 µm LbCas12a endonuclease, 5 nm TcrRNA‐HBV, 10 nm HBV‐Fg, 50 nm CcrRNA, 50 nm circular single‐stranded DNA (ssDNA) substrate, 50 nm chimeric circular crRNA targeting EGFP, 0.5 U/µL RNase inhibitor, and nuclease‐free water to a total volume of 30 µL. Reactions were incubated at 37°C for 1 h. Following incubation, the reaction products were resolved by agarose gel electrophoresis to assess cleavage and gene editing efficiency by the presence of specific cleavage bands compared to controls.

### 4.7 In Vivo Validation of Conditional Gene Editing Using the TopCas System

A549‐EGFP‐Puro cells (kindly provided by Dr. Huang Zhen's laboratory) were seeded at a density of 1.0 × 10^6 cells per well in 6‐well plates and incubated overnight. Cells were transfected with plasmids encoding hepatitis B virus (HBV) genome fragments using Lipofectamine 3000 according to the manufacturer's protocol. After 24 h, to allow expression of target nucleic acids, cells were transfected with the HBV‐specific TopCas system, which contained a mixture of chimeric circular crRNAs—one designed to initiate self‐catalytic activation and two targeting distinct sites within the EGFP gene. The TopCas complexes were preassembled in vitro and transfected into the HBV‐transfected A549‐EGFP‐Puro cells using Lipofectamine CRISPRMAX reagent (Thermo Fisher Scientific). Cells were cultured for an additional 48 h post‐transfection. EGFP expression was assessed by laser scanning confocal microscopy (ZEISS LSM900) and flow cytometry (FACSymphony A1) to evaluate gene editing efficiency at the protein level. For genotypic analysis, genomic DNA was extracted from cells using a DNA extraction kit (OMEGA). To enrich for edited alleles, the extracted DNA was treated with Cas9 nuclease (NEB) targeting the unedited EGFP sequence, thereby selectively cleaving wild‐type alleles. The edited gene fragments were subsequently PCR amplified and purified using a PCR purification kit (OMEGA). Gene editing efficiency was further analyzed using a mutation detection kit (T7 Endonuclease I assay, New England Biolabs) following the manufacturer's instructions. Reaction products were resolved by agarose gel electrophoresis, and cleavage band intensities were quantified to determine editing frequencies.

### 4.8 Statistical Analysis

Data is shown as mean ± SD, with dots representing individual donors (average of technical duplicates, *n* = 3). Statistical differences between groups were determined using one‐way ANOVA with Tukey post‐tests. *p* < 0.05 (*); *p* < 0.01 (**); *p* < 0.001 (***); *p* < 0.0001 (****); ns: not significant. Statistical analyses were performed using GraphPad Prism 9 (Graphpad Software). Specific statistical tests are annotated within the respective figure legends.

## Funding

The National Science and Technology Major Project on the Prevention and Treatment of Cancers, Cardiovascular and Cerebrovascular Diseases, Respiratory and Metabolic Diseases (2025ZD0551200); the National Key Research and Development Program of China (2022YFC2603800).

## Conflicts of Interest

The authors declare no conflict of interest.

## Supporting information




**Supporting File**: advs75046‐sup‐0001‐SuppMat.docx.

## Data Availability

The data that support the findings of this study are available in the supplementary material of this article.
