# Supplementary material for: TopCas: Topology‐Gated Cas12a via DNA‐RNA Chimeric Circular crRNA for Amplification‐Free Nucleic Acid Detection and Conditional Gene Editing
Source: Adv Sci (Weinh). 2026 Mar 30;13(33):e75046. doi: 10.1002/advs.75046 (PMC13271592; doi:10.1002/advs.75046)
Supplement: Supplementary file 1 — Supporting File: advs75046‐sup‐0001‐SuppMat.docx. [file ADVS-13-e75046-s001.docx]

Supporting Information

TopCas: Topology-Gated Cas12a via DNA-RNA Chimeric Circular crRNA for Amplification-Free Nucleic Acid Detection and Conditional Gene Editing

Shun Zhang^#^, Wen Sun^#^, Ting Xiao, Yali Wang, Xianlan Wu, Huiyou Chen*, Ming Chen* and Jun Zhang*

S. Zhang, T. Xiao, X. Wu, H. Chen, M. Chen, J. Zhang

Department of Clinical Laboratory Medicine, Southwest Hospital
Third Military Medical University (Army Medical University)
30 Gaotanyan, Shapingba District, Chongqing 400038 (P. R. China)
E-mail: g1andor@tmmu.edu.cn; chenming1971@tmmu.edu.cn; xnzj@tmmu.edu.cn

W. Sun, Y. Wang
Department Key Laboratory of Bio-Resource and Eco-environment of Ministry of Education College of Life Sciences. Sichuan University
Chengdu, Sichuan 610065 (P. R. China)

Experimental Section

*Urea-PAGE Analysis of Circular crRNA and Cas12a Trans-cleavage Products:* The formation of circular crRNA and Cas12a-mediated trans-cleavage products was analyzed by denaturing urea polyacrylamide gel electrophoresis (Urea-PAGE). For analysis, 2 µL of the circular crRNA reaction product was mixed with 3 µL nuclease-free water and 5 µL of 2× Urea-gel loading dye (containing 7 M urea and tracking dyes). Samples were denatured at 95°C for 5 minutes prior to loading. Samples were electrophoresed on a preheated 16% TBE-Urea gel (8 M urea) at 55°C for 30 minutes under a constant voltage of 180 V. Gels were subsequently stained and visualized using the Gel Doc+ XR imaging system (Bio-Rad Laboratories, USA). Cas12a trans-cleavage products were analyzed following the same procedure.

*Cas12a Trans-cleavage Activity Assay:* A standard CRISPR/Cas12a 30 μL reaction contained 1 µL of 1 µM LbCas12a endonuclease (New England Biolabs), 2 µL of 0.5 µM single guide RNA (crRNA; different types), 3 μL of 10× NEBuffer r2.1 reaction buffer, 3 μL of 10 µM ssDNA reporter (5′-FAM-ssDNA-BHQ1-3′ or FAM-ssDNA), 0.1 μM substrate DNA, 0.5 U/μL RNase inhibitor and nuclease-free water to volume. The mixture was thoroughly mixed and incubated at room temperature for 60 minutes in a thermal cycler. Following the reaction, cleavage products were analyzed either by urea-PAGE or by measuring fluorescence intensity using a SpectraMax microplate reader with excitation and emission wavelengths set to 494 nm and 519 nm.

In this study, various types of crRNAs, substrates, and reporter molecules were systematically analyzed. The crRNAs included linear native crRNA, linear chimeric crRNA, circular native crRNA, and circular chimeric crRNA. Substrates tested encompassed linear single-stranded DNA (ssDNA), linear double-stranded DNA (dsDNA), circular ssDNA, and phosphorothioate-modified substrates. The reporter molecules consisted of circular chimeric crRNA and fluorophore-quencher oligonucleotides with different sequences. For each reaction, only the component under investigation was varied, while all other reaction conditions and components were kept constant to ensure comparability.

*Circular Topology Validation via DNA Tetrahedron Assembly and Dot Blot Hybridization Analysis:* Circular Topology Validation via DNA Tetrahedron Assembly: Single-stranded linear and circular DNA oligonucleotides designed to self-assemble into tetrahedral nanostructures were prepared at equimolar concentrations. Assembly reactions were performed in assembly buffer consisting of 10 mM Tris-HCl (pH 8.0), 50 mM NaCl, and 10 mM MgCl₂. Samples were heated to 95°C for 5 minutes and then slowly cooled to 4°C over a 2-hour period to facilitate proper folding and hybridization. Successful formation of higher-order tetrahedral structures was confirmed by native 6% TBE-PAGE under non-denaturing conditions, followed by staining with Ethidium bromide (EB) and visualization using a Gel Doc+ XR imaging system.

*Dot Blotting Assay:* The dot blot assay was performed on nitrocellulose membranes (Millipore). After marking the sample application sites, 2 µL of Cas12a protein (1 µM) was spotted onto each test zone. The membrane was dried at 37°C for 30 minutes, then blocked overnight at 4°C with 5% skimmed milk prepared in 1× TBST (Tris-buffered saline with 0.1% Tween-20). Following blocking, the membrane was washed three times with 1× TBST. Subsequently, 2 µL of 20 µM FAM-labeled linear crRNA (LcrRNA) or circular crRNA (CcrRNA) was applied to each test zone and incubated for 30 minutes at room temperature. The membrane was then placed in a trough containing 1× TBST and subjected to gentle agitation for 5 minutes. This washing step was repeated three times. After washing, the membrane was air-dried at room temperature. Fluorescence intensity at each dot was scanned and quantified using the Gel Doc+ XR imaging system. The assay was also performed similarly using CcrRNA as the binding molecule, paired respectively with FAM-labeled linear DNA substrates and FAM-labeled circular DNA substrates, while all other conditions remained unchanged.

*Linearization of Chimeric Circular crRNA by Activated Cas12a and Activity Assessment:* To evaluate linearization of chimeric circular crRNA by activated Cas12a, the standard Cas12a trans-cleavage assay was modified by replacing the single-stranded DNA (ssDNA) substrate with chimeric circular crRNA containing a 5–15 nt DNA linker segment. Following completion of the cleavage reaction, nucleic acids were extracted using phenol: chloroform: isoamyl alcohol (25:24:1, v/v/v) to remove protein contaminants. The aqueous phase was subsequently treated with Exonuclease III and Exonuclease I at 37°C for 1 hour to digest residual linear DNA. The enzymatic reaction was terminated by heating at 95°C for 5 minutes. The purified, potentially linearized crRNA product was then used as the guide RNA in a fresh Cas12a cleavage reaction, together with the corresponding substrate and a FAM-labeled ssDNA reporter molecule. Activation of Cas12a nuclease activity was monitored by assessing cleavage of the FAM-ssDNA reporter. Integrity and cleavage of the reporter were analyzed by denaturing urea-PAGE, allowing evaluation of Cas12a activity as indicated by the disappearance of intact FAM-ssDNA.

Table S1. DNA sequences for this study.

| Name | Sequence (5′-3′) | Figure |
| --- | --- | --- |
| ATCG | P-(A,T,C,G)GATCAAGCTCAGTGAGTAAGGCACGTCAA(A,T,C,G) | 2D |
| Liner substrates 1 | AGCTCAGTGAGTAAGGCACGTCAAA | 3C, S13 |
| FAM-ssDNA | FAM-GTCGAGTCAAGAGCATCC | 3, 4A-B, S5, S13 |
| Liner substrates 2 | GAGCACCCAGTCCGCCCTGAG | 4B-E,5A-F |
| F-Q | FAM-TTATTATTA-BHQ1 | 4D-E,5,6 |
| S | A*G*C*T*C*A*G*T*G*A*G*T*A*A*G*G*C*A*C*G*T*C*A*A*A | 5B |
| sC | A*G*C*T*C*AGTGAGTAAGGCACGTCAAA | 5B |
| sP | AGCTCAGTGAGTAAGGCACGT*C*A*A*A | 5B-J,6A-E,7 |
| sA | AGCTC*AGTG*AGT*A*AGGCACGTC*A*A*A | S8 |
| sT | AGC*TCAG*TGAG*TAAGGCACG*TCAAA | S8 |
| sC | AG*CT*CAGTGAGTAAGG*CA*CGT*CAAA | S8 |
| sG | A*GCTCA*GT*GA*GTAA*G*GCAC*GTCAAA | S8 |
| sM | AGCTCAGTG*A*G*T*A*A*G*G*CACGTCAAA | S8 |
| sDC | ds-AGCTCAGTGAGTAAGGCACGTCAAA | S8 |
| Cy5-Q | Cy5-TTATTATTA-BHQ3 | 6E |
| EGFP-F | CAAATGGTGAGCAAGGGCGA | 7B,7G |
| EGFP-R | GCCACTTGTGTAGCGCCA | 7B,7G |
| s20 | AGCACCCAGTCCGCCCTGAG | S5 |
| s40 | CCACTACCTGAGCACCCAGTCCGCCCTGAGCAAAGACCCC | S5 |
| s80 | CCCCGTGCTGCTGCCCGACAACCACTACCTGAGCACCCAGTCCGCCCTGAGCAAAGACCCCAACGAGAAGCGCGATCACA | S5 |
| s100 | CGGCGACGGCCCCGTGCTGCTGCCCGACAACCACTACCTGAGCACCCAGTCCGCCCTGAGCAAAGACCCCAACGAGAAGCGCGATCACATGGTCCTGCTG | S5 |
| d20 | ds-AGCACCCAGTCCGCCCTGAG | S5 |
| d40 | ds-CCACTACCTGAGCACCCAGTCCGCCCTGAGCAAAGACCCC | S5 |
| d80 | ds-CCCCGTGCTGCTGCCCGACAACCACTACCTGAGCACCCAGTCCGCCCTGAGCAAAGACCCCAACGAGAAGCGCGATCACA | S5 |
| d200 | ds-GAGGACGGCAGCGTGCAGCTCGCCGACCACTACCAGCAGAACACCCCCATCGGCGACGGCCCCGTGCTGCTGCCCGACAACCACTACCTGAGCACCCAGTCCGCCCTGAGCAAAGACCCCAACGAGAAGCGCGATCACATGGTCCTGCTGGAGTTCGTGACCGCCGCCGGGATCACTCTCGGCATGGACGAGCTGTACAA | S5 |
| DNA_FAM_ 1 | FAM-AGCTCAGTGAGTAAGGCACGTCAAA | S6 |
| DNA_FAM_ 2 | P-AGCTCAGTGAGTA_FAM_AGGCACGTCAAA | S6 |
| L1 | GTGTTGTCATGCATTATTACTGCTGACAAATCAAGAGATTATTAATCCA | S7 |
| L2 | GCAGATTATTAATTAGGTGTTGAAGAGACATTATTACTCTTGATTTGTCA | S7 |
| L3 | GTCTCTTCAACACCTAATATTATTATCAGACTGCTCTTTCATAATTATTA | S7 |
| L4 | GAGCAGTCTGAATTATTAGCATGACAACACTGGATATTATTATATGAAA | S7 |
| EGFP-Fg | GACAACCACTACCTGAGCACCCAGTCCGCCCTGAGCAAAGACCCCAACGA | S16 |
| HBV-Fg | CCAAACAGTGGGGGAAAGCCCTACGAACCACTGAACAAATGGCACTAGTA | 7B, S16 |
| HIV-Fg | TGCTAAACACAGTAGGAGGACATCAAGCAGCCATGCAAATGTTAAAAGAG | S16 |
| E-CcrRNA-1 | -GGATTATTA**TCCGCCCTGAG**TAA- | 7B-E |
| E-CcrRNA-2 | -GGATTATTA**GGACGGCGACG**TAA- | 7B-E |
| pLVX-Puro | tggaagggctaattcactcccaaagaagacaagatatccttgatctgtggatctaccacacacaaggctacttccctgattagcagaactacacaccagggccaggggtcagatatccactgacctttggatggtgctacaagctagtaccagttgagccagataaggtagaagaggccaataaaggagagaacaccagcttgttacaccctgtgagcctgcatgggatggatgacccggagagagaagtgttagagtggaggtttgacagccgcctagcatttcatcacgtggcccgagagctgcatccggagtacttcaagaactgctgatatcgagcttgctacaagggactttccgctggggactttccagggaggcgtggcctgggcgggactggggagtggcgagccctcagatcctgcatataagcagctgctttttgcctgtactgggtctctctggttagaccagatctgagcctgggagctctctggctaactagggaacccactgcttaagcctcaataaagcttgccttgagtgcttcaagtagtgtgtgcccgtctgttgtgtgactctggtaactagagatccctcagacccttttagtcagtgtggaaaatctctagcagtggcgcccgaacagggacttgaaagcgaaagggaaaccagaggagctctctcgacgcaggactcggcttgctgaagcgcgcacggcaagaggcgaggggcggcgactggtgagtacgccaaaaattttgactagcggaggctagaaggagagagatgggtgcgagagcgtcagtattaagcgggggagaattagatcgcgatgggaaaaaattcggttaaggccagggggaaagaaaaaatataaattaaaacatatagtatgggcaagcagggagctagaacgattcgcagttaatcctggcctgttagaaacatcagaaggctgtagacaaatactgggacagctacaaccatcccttcagacaggatcagaagaacttagatcattatataatacagtagcaaccctctattgtgtgcatcaaaggatagagataaaagacaccaaggaagctttagacaagatagaggaagagcaaaacaaaagtaagaccaccgcacagcaagcggccggccgctgatcttcagacctggaggaggagatatgagggacaattggagaagtgaattatataaatataaagtagtaaaaattgaaccattaggagtagcacccaccaaggcaaagagaagagtggtgcagagagaaaaaagagcagtgggaataggagctttgttccttgggttcttgggagcagcaggaagcactatgggcgcagcgtcaatgacgctgacggtacaggccagacaattattgtctggtatagtgcagcagcagaacaatttgctgagggctattgaggcgcaacagcatctgttgcaactcacagtctggggcatcaagcagctccaggcaagaatcctggctgtggaaagatacctaaaggatcaacagctcctggggatttggggttgctctggaaaactcatttgcaccactgctgtgccttggaatgctagttggagtaataaatctctggaacagatttggaatcacacgacctggatggagtgggacagagaaattaacaattacacaagcttaatacactccttaattgaagaatcgcaaaaccagcaagaaaagaatgaacaagaattattggaattagataaatgggcaagtttgtggaattggtttaacataacaaattggctgtggtatataaaattattcataatgatagtaggaggcttggtaggtttaagaatagtttttgctgtactttctatagtgaatagagttaggcagggatattcaccattatcgtttcagacccacctcccaaccccgaggggacccgacaggcccgaaggaatagaagaagaaggtggagagagagacagagacagatccattcgattagtgaacggatctcgacggtatcgcctttaaaagaaaaggggggattggggggtacagtgcaggggaaagaatagtagacataatagcaacagacatacaaactaaagaattacaaaaacaaattacaaaaattcaaaattttcgggtttattacagggacagcagagatccagtttatcgataagcttgggagttccgcgttacataacttacggtaaatggcccgcctggctgaccgcccaacgacccccgcccattgacgtcaataatgacgtatgttcccatagtaacgccaatagggactttccattgacgtcaatgggtggagtatttacggtaaactgcccacttggcagtacatcaagtgtatcatatgccaagtacgccccctattgacgtcaatgacggtaaatggcccgcctggcattatgcccagtacatgaccttatgggactttcctacttggcagtacatctacgtattagtcatcgctattaccatggtgatgcggttttggcagtacatcaatgggcgtggatagcggtttgactcacggggatttccaagtctccaccccattgacgtcaatgggagtttgttttggcaccaaaatcaacgggactttccaaaatgtcgtaacaactccgccccattgacgcaaatgggcggtaggcgtgtacggtgggaggtctatataagcagagctcgtttagtgaaccgtcagatcgcctggagacgccatccacgctgttttgacctccatagaagacaccgactctactagaggatcgctagcgctaccggactcagatctcgagctcaaggatcccgcgactctagataattctaccgggtaggggaggcgcttttcccaaggcagtctggagcatgcgctttagcagccccgctgggcacttggcgctacacaagtggcctctggcctcgcacacattccacatccaccggtaggcgccaaccggctccgttctttggtggccccttcgcgccaccttctactcctcccctagtcaggaagttcccccccgccccgcagctcgcgtcgtgcaggacgtgacaaatggaagtagcacgtctcactagtctcgtgcagatggacagcaccgctgagcaatggaagcgggtaggcctttggggcagcggccaatagcagctttgctccttcgctttctgggctcagaggctgggaaggggtgggtccgggggcgggctcaggggcgggctcaggggcggggcgggcgcccgaaggtcctccggaggcccggcattctgcacgcttcaaaagcgcacgtctgccgcgctgttctcctcttcctcatctccgggcctttcgacctgcagcccaagcttaccatgaccgagtacaagcccacggtgcgcctcgccacccgcgacgacgtccccagggccgtacgcaccctcgccgccgcgttcgccgactaccccgccacgcgccacaccgtcgatccggaccgccacatcgagcgggtcaccgagctgcaagaactcttcctcacgcgcgtcgggctcgacatcggcaaggtgtgggtcgcggacgacggcgccgcggtggcggtctggaccacgccggagagcgtcgaagcgggggcggtgttcgccgagatcggcccgcgcatggccgagttgagcggttcccggctggccgcgcagcaacagatggaaggcctcctggcgccgcaccggcccaaggagcccgcgtggttcctggccaccgtcggcgtctcgcccgaccaccagggcaagggtctgggcagcgccgtcgtgctccccggagtggaggcggccgagcgcgccggggtgcccgccttcctggagacctccgcgccccgcaacctccccttctacgagcggctcggcttcaccgtcaccgccgacgtcgaggtgcccgaaggaccgcgcacctggtgcatgacccgcaagcccggtgcctgaccgcgtctggaacaatcaacctctggattacaaaatttgtgaaagattgactggtattcttaactatgttgctccttttacgctatgtggatacgctgctttaatgcctttgtatcatgctattgcttcccgtatggctttcattttctcctccttgtataaatcctggttgctgtctctttatgaggagttgtggcccgttgtcaggcaacgtggcgtggtgtgcactgtgtttgctgacgcaacccccactggttggggcattgccaccacctgtcagctcctttccgggactttcgctttccccctccctattgccacggcggaactcatcgccgcctgccttgcccgctgctggacaggggctcggctgttgggcactgacaattccgtggtgttgtcggggaagctgacgtcctttccatggctgctcgcctgtgttgccacctggattctgcgcgggacgtccttctgctacgtcccttcggccctcaatccagcggaccttccttcccgcggcctgctgccggctctgcggcctcttccgcgtcttcgccttcgccctcagacgagtcggatctccctttgggccgcctccccgcctggaattaattctgcagtcgagacctagaaaaacatggagcaatcacaagtagcaatacagcagctaccaatgctgattgtgcctggctagaagcacaagaggaggaggaggtgggttttccagtcacacctcaggtacctttaagaccaatgacttacaaggcagctgtagatcttagccactttttaaaagaaaagaggggactggaagggctaattcactcccaacgaagacaagatatccttgatctgtggatctaccacacacaaggctacttccctgattagcagaactacacaccagggccaggggtcagatatccactgacctttggatggtgctacaagctagtaccagttgagccagataaggtagaagaggccaataaaggagagaacaccagcttgttacaccctgtgagcctgcatgggatggatgacccggagagagaagtgttagagtggaggtttgacagccgcctagcatttcatcacgtggcccgagagctgcatccggagtacttcaagaactgctgatatcgagcttgctacaagggactttccgctggggactttccagggaggcgtggcctgggcgggactggggagtggcgagccctcagatcctgcatataagcagctgctttttgcctgtactgggtctctctggttagaccagatctgagcctgggagctctctggctaactagggaacccactgcttaagcctcaataaagcttgccttgagtgcttcaagtagtgtgtgcccgtctgttgtgtgactctggtaactagagatccctcagacccttttagtcagtgtggaaaatctctagcagtagtagttcatgtcatcttattattcagtatttataacttgcaaagaaatgaatatcagagagtgagaggccttgacattgctagcgttttaccgtcgacctctagctagagcttggcgtaatcatggtcatagctgtttcctgtgtgaaattgttatccgctcacaattccacacaacatacgagccggaagcataaagtgtaaagcctggggtgcctaatgagtgagctaactcacattaattgcgttgcgctcactgcccgctttccagtcgggaaacctgtcgtgccagctgcattaatgaatcggccaacgcgcggggagaggcggtttgcgtattgggcgctcttccgcttcctcgctcactgactcgctgcgctcggtcgttcggctgcggcgagcggtatcagctcactcaaaggcggtaatacggttatccacagaatcaggggataacgcaggaaagaacatgtgagcaaaaggccagcaaaaggccaggaaccgtaaaaaggccgcgttgctggcgtttttccataggctccgcccccctgacgagcatcacaaaaatcgacgctcaagtcagaggtggcgaaacccgacaggactataaagataccaggcgtttccccctggaagctccctcgtgcgctctcctgttccgaccctgccgcttaccggatacctgtccgcctttctcccttcgggaagcgtggcgctttctcatagctcacgctgtaggtatctcagttcggtgtaggtcgttcgctccaagctgggctgtgtgcacgaaccccccgttcagcccgaccgctgcgccttatccggtaactatcgtcttgagtccaacccggtaagacacgacttatcgccactggcagcagccactggtaacaggattagcagagcgaggtatgtaggcggtgctacagagttcttgaagtggtggcctaactacggctacactagaagaacagtatttggtatctgcgctctgctgaagccagttaccttcggaaaaagagttggtagctcttgatccggcaaacaaaccaccgctggtagcggtggtttttttgtttgcaagcagcagattacgcgcagaaaaaaaggatctcaagaagatcctttgatcttttctacggggtctgacgctcagtggaacgaaaactcacgttaagggattttggtcatgagattatcaaaaaggatcttcacctagatccttttaaattaaaaatgaagttttaaatcaatctaaagtatatatgagtaaacttggtctgacagttaccaatgcttaatcagtgaggcacctatctcagcgatctgtctatttcgttcatccatagttgcctgactccccgtcgtgtagataactacgatacgggagggcttaccatctggccccagtgctgcaatgataccgcgagacccacgctcaccggctccagatttatcagcaataaaccagccagccggaagggccgagcgcagaagtggtcctgcaactttatccgcctccatccagtctattaattgttgccgggaagctagagtaagtagttcgccagttaatagtttgcgcaacgttgttgccattgctacaggcatcgtggtgtcacgctcgtcgtttggtatggcttcattcagctccggttcccaacgatcaaggcgagttacatgatcccccatgttgtgcaaaaaagcggttagctccttcggtcctccgatcgttgtcagaagtaagttggccgcagtgttatcactcatggttatggcagcactgcataattctcttactgtcatgccatccgtaagatgcttttctgtgactggtgagtactcaaccaagtcattctgagaatagtgtatgcggcgaccgagttgctcttgcccggcgtcaatacgggataataccgcgccacatagcagaactttaaaagtgctcatcattggaaaacgttcttcggggcgaaaactctcaaggatcttaccgctgttgagatccagttcgatgtaacccactcgtgcacccaactgatcttcagcatcttttactttcaccagcgtttctgggtgagcaaaaacaggaaggcaaaatgccgcaaaaaagggaataagggcgacacggaaatgttgaatactcatactcttcctttttcaatattattgaagcatttatcagggttattgtctcatgagcggatacatatttgaatgtatttagaaaaataaacaaataggggttccgcgcacatttccccgaaaagtgccacctgacgtcgacggatcgggagatcaacttgtttattgcagcttataatggttacaaataaagcaatagcatcacaaatttcacaaataaagcatttttttcactgcattctagttgtggtttgtccaaactcatcaatgtatcttatcatgtctggatcaactggataactcaagctaaccaaaatcatcccaaacttcccaccccataccctattaccactgccaattacctgtggtttcatttactctaaacctgtgattcctctgaattattttcattttaaagaaattgtatttgttaaatatgtactacaaacttagtagtttttaaagaaattgtatttgttaaatatgtactacaaacttagtagt | 6D,7E |

The DNA sequences utilized in this study are listed above, where "P" denotes 5' phosphorylation, "*" indicates phosphorothioate modification, and "ds" represents double-stranded DNA.

Table S2. RNA sequences for this study.

| Name | Sequence (5′-3′) | Figure |
| --- | --- | --- |
| crRNA | P-GUAAUUUCUACUAAGUGUAGAUACGUGCCUUACUCACUGAGCUUGA | 2A, S2-4 |
| Liner crRNA 1 | GUAAUUUCUACUAAGUGUAGAUACGUGCCUUACUCACUGAGCUUGA | 3A-B |
| -2 crRNA | AAUUUCUACUAAGUGUAGAUACGUGCCUUACUCACUGAGCUUGA | 4A |
| -4 crRNA | UUUCUACUAAGUGUAGAUACGUGCCUUACUCACUGAGCUUGA | 4A |
| -6 crRNA | UCUACUAAGUGUAGAUACGUGCCUUACUCACUGAGCUUGA | 4A |
| 11 crRNA | GUAAUUUCUACUAAGUGUAGAUACGUGCCUUAC | 4A |
| 13 crRNA | GUAAUUUCUACUAAGUGUAGAUACGUGCCUUACUC | 4A |
| 15 crRNA | GUAAUUUCUACUAAGUGUAGAUACGUGCCUUACUCAC | 4A |
| 17 crRNA | GUAAUUUCUACUAAGUGUAGAUACGUGCCUUACUCACUG | 4A |
| Liner crRNA 2 | GUAAUUUCUACUAAGUGUAGAUCUCAGGGCGGACUGGGUGCUC | 4B-E, 5A-F-I-J, |
| HPV16 crRNA | GUAAUUUCUACUAAGUGUAGAUGUGCUAUGGACUUUACUACA | 6A |
| SARS-CoV-2 crRNA | GUAAUUUCUACUAAGUGUAGAUUUGAACUGUUGCGACUACGUGA | 6B |
| HBV crRNA | GUAAUUUCUACUAAGUGUAGAUUUCAGUGGUUCGUAGGGCUU | 5G-H, 6E, 7B-E |
| HIV crRNA | GUAAUUUCUACUAAGUGUAGAUCAUGGCUGCUUGAUGUCCUC | 5G-H, S13 |
| EGFP-crRNA 1 | GUAAUUUCUACUAAGUGUAGAUCUCAGGGCGGACUGGGUGCUC | 5G-H, |
| crRNA-FAM | P-GUAAUUUCUACUAAGUGUAGAU_FAM_ACGUGCCUUACUCACUGAGCUUGA | S6 |
| RNA substrates | GAGGACAUCAAGCAGCCAUGC | S13 |

The DNA sequences utilized in this study are listed above, where "P" denotes 5' phosphorylation.

Table S3. DNA-RNA chimeric sequences for this study.

| Name | Sequence (5′-3′) | Figure |
| --- | --- | --- |
| 5 nt | P-GAUACGUGCCUUACUCACUGAGC***TTATT***GUAAUUUCUACUAAGUGUA | 2A-B,3C,4B-D,5A |
| 10 nt | P-GAUACGUGCCUUACUCACUGAGC***TTATTATTAT***GUAAUUUCUACUAAGUGUA | 2B,3C-D,4B-D |
| PS-10 nt | P-GAUACGUGCCUUACUCACUGAG*C****TTATTATTAT****G*UAAUUUCUACUAAGUGUA | S12,5A-J,6A-E,7 |
| 15 nt | P-GAUACGUGCCUUACUCACUGAGC***TTATTATTATTATTA***GUAAUUUCUACUAAGUGUA | 2B,3C,4B-D, 5A |
| AUCG | P-(A,U,C,G)AUACGUGCCUUACUCACUGAGC***TTATTATTAT***GUAAUUUCUACUAAGUGU(A,U,C,G) | 2C |
| Linker 20 | P-GAUACGUGCCUUACUCACUGAGC***TTATTATTATTATTATTATT***GUAAUUUCUACUAAGUGUA | 3C |
| Linker 25 | P-GAUACGUGCCUUACUCACUGAGC***TTATTATTATTATTATTATTATTAT***GUAAUUUCUACUAAGUGUA | 3C |
| Linker 30 | P-GAUACGUGCCUUACUCACUGAGC***TTATTATTATTATTATTATTATTATTATTA***GUAAUUUCUACUAAGUGUA | 3C |
| Linker 40 | P-GAUACGUGCCUUACUCACUGAGC***TTATTATTATTATTATTATTATTATTATTATTATTATTAT***GUAAUUUCUACUAAGUGUA | 3C |
| EGFP-crRNA 1 | P-GAU**CUCAGGGCGGA**CUGGGUGCUC***TTATTATTAT***GUAAUUUCUACUAAGUGUA | 7B-E |
| EGFP-crRNA 2 | P-GAU**CGUCGCCGUCC**AGCUCGACC***TTATTATTAT***GUAAUUUCUACUAAGUGUA | 7B-E, S17 |
| 1 | P-GAUACGUGCCUUACUCACUGAGC***TTATTATTAT***GUAAUUUCUACUAAGUGUA | S11 |
| 2 | GAUACGUGCCUUACUCACUGAGC***TTATTATTAT***GUAAUUUCUACUAAGUGUA | S11 |
| 3 | AGUGUAGAUACGUGCCUUACUCACUGAGC***TTATTATTAT***GUAAUUUCUACUA | S11 |
| 4 | UCUACUAAGUGUAGAUACGUGCCUUACUCACUGAGC***TTATTATTAT***GUAAUU | S11 |
| 5 | CUCACUGAGC***TTATTATTAT***GUAAUUUCUACUAAGUGUAGAUACGUGCCUUA | S11 |
| FQ-crRNA | P-GAUACGUGCCUUACUCACUGAGC_FAM_***TTATTATTAT***_BHQ1_GUAAUUUCUACUAAGUGUA | S15 |

# The DNA-RNA chimeric sequences utilized in this study are listed above, where "P" denotes 5' phosphorylation, "*Bold italic type*" denotes the DNA portion.

Table S4. The clinical nucleic acid samples of HPV and SARS-CoV-2.

|  | 1 | 2 | 3 | 4 | 5 | 6 | 7 | 8 | 9 | 10 |
| --- | --- | --- | --- | --- | --- | --- | --- | --- | --- | --- |
| HPV16**+** | **+** | **+** | **+** | **+** | **+** | **+** | **+** | **+** | **+** | **+** |
| HPV16- | **-** | **-** | **-** | **-** | **-** | **-** | **-** | **-** | **-** | **-** |
| SARS-CoV-2 **+** | **+** | **+** | **+** | **+** | **+** | **+** | **+** | **+** | **+** | **+** |
| SARS-CoV-2 **-** | **-** | **-** | **-** | **-** | **-** | **-** | **-** | **-** | **-** | **-** |

The inclusion criteria for these specimens were based on clinical positive/negative diagnostic standards; therefore, precise PCR Ct values are not available. Among the HPV16 cases, the cohort comprised both mono-infections and co-infections. Sample identifiers denote ordinal numbers only and do not refer to the same specimen across measurements.

Supplementary figures

**
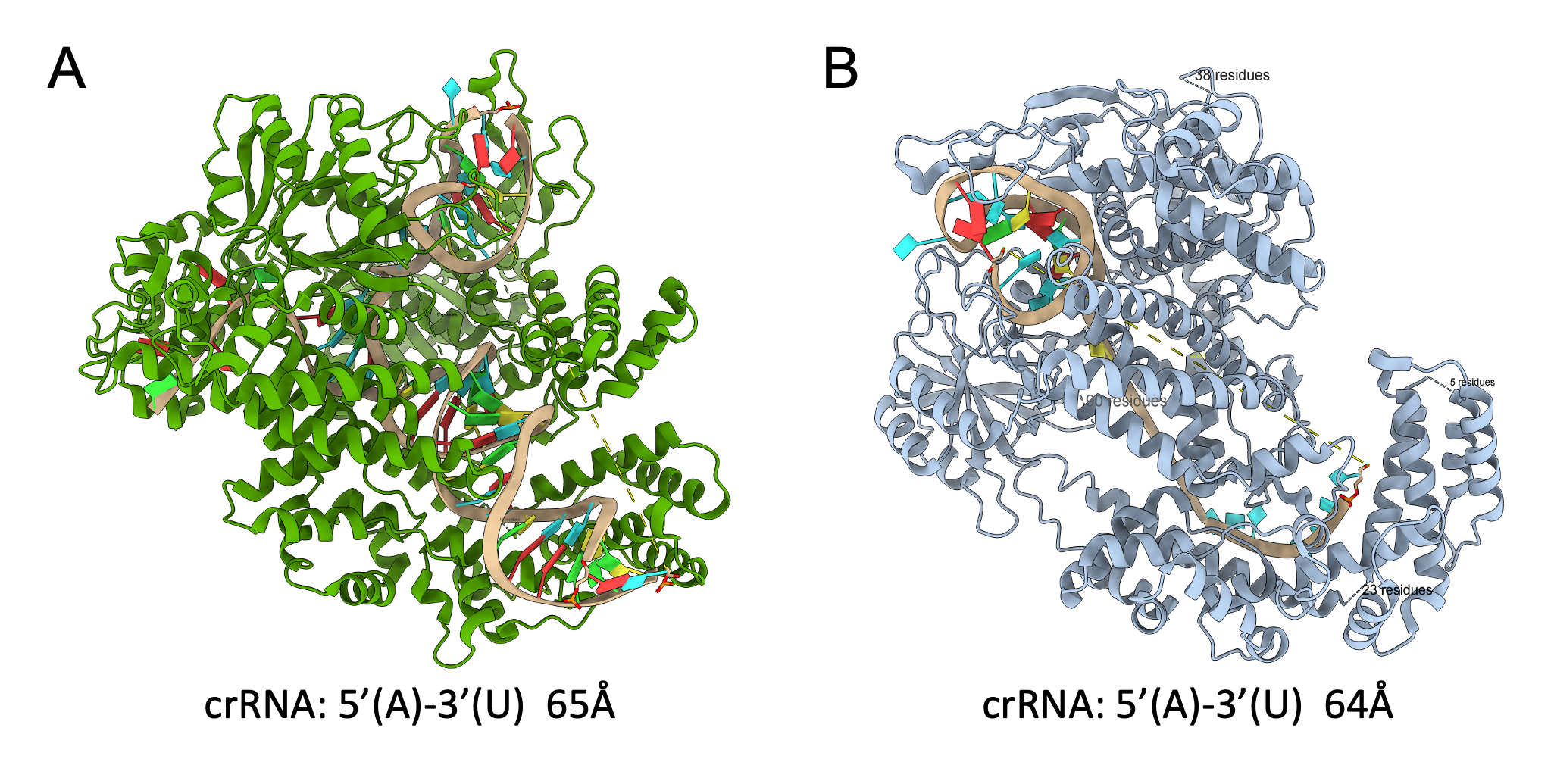
**

**Figure S1.** Structure of the Cas12a protein in complex with crRNA. (A) The distance between crRNA 5’-3’ bases in the Cas12a-DNA-crRNA complex (PDB: 8D4A). (B) The distance between crRNA 5’-3’ bases in the Cas12a-crRNA complex (PDB: 8D49).

**
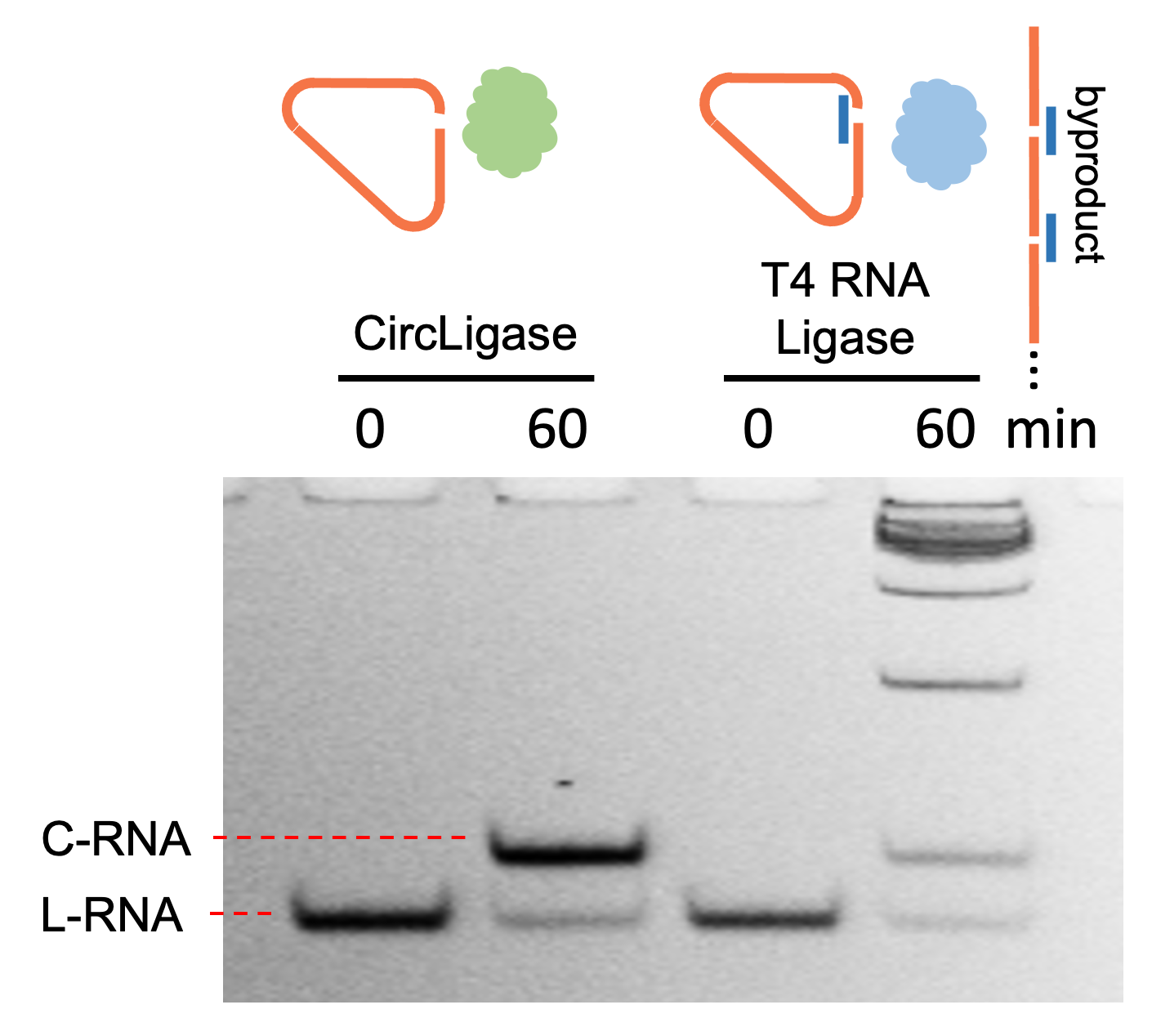
**

**Figure S2.** Comparison of RNA circularization methods. Lane 1: The liner RNA substrate. Lane 2: The product of CircLigase reaction. Lane 3: The liner RNA substrate. Lane 4: T4 RNA ligase reaction products after exonuclease treatment. The experiments were conducted in three technical replicates.


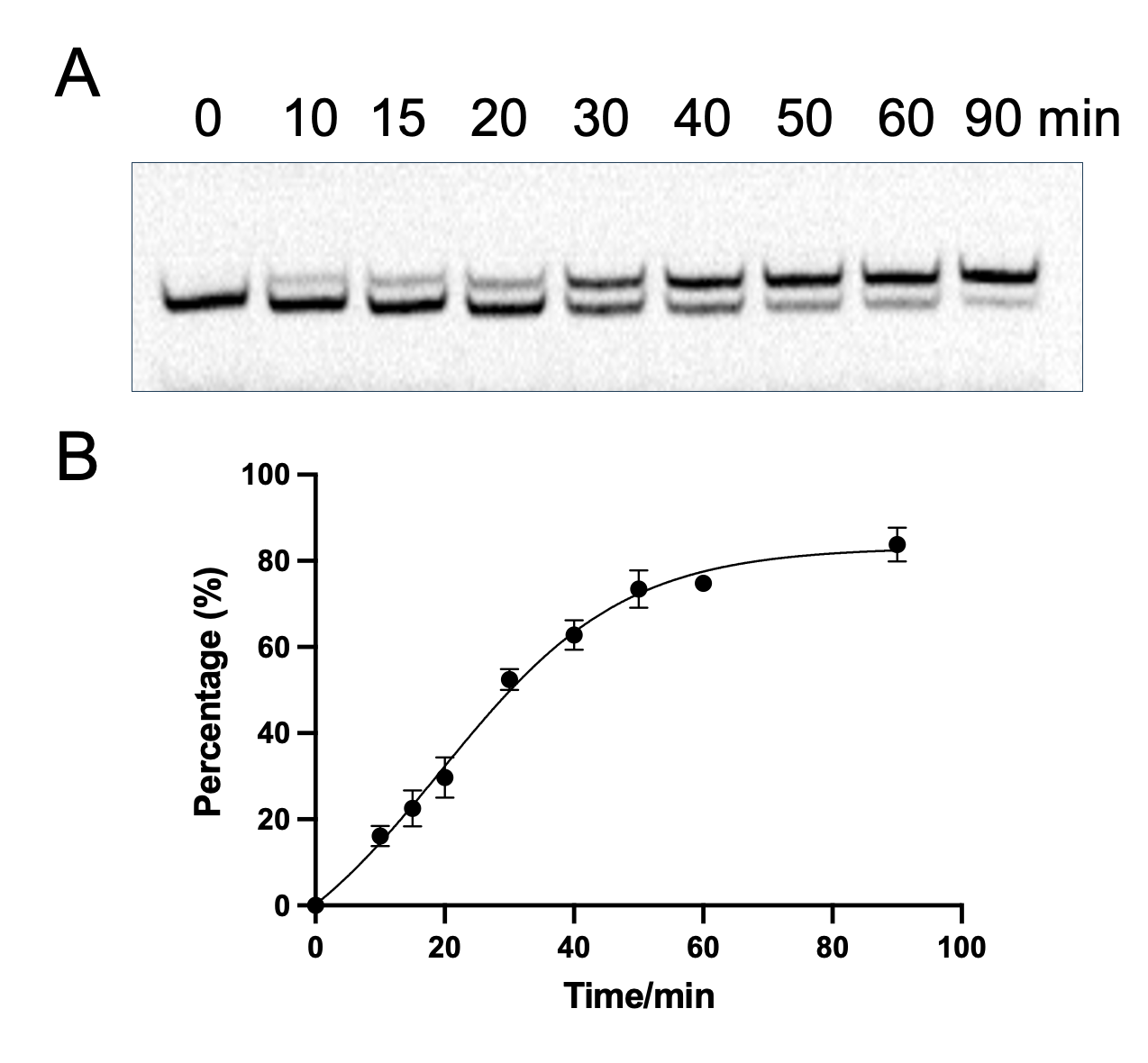


**Figure S3.** Reaction time course of CircLigase-mediated circularization (46 nt; 5′G–3′A). (A) Denaturing urea-PAGE analysis of reaction products at different time points. (B) Quantification of band intensities from urea-PAGE gels by ImageJ. Data represent the mean ± SD from three technical replicates (n = 3).


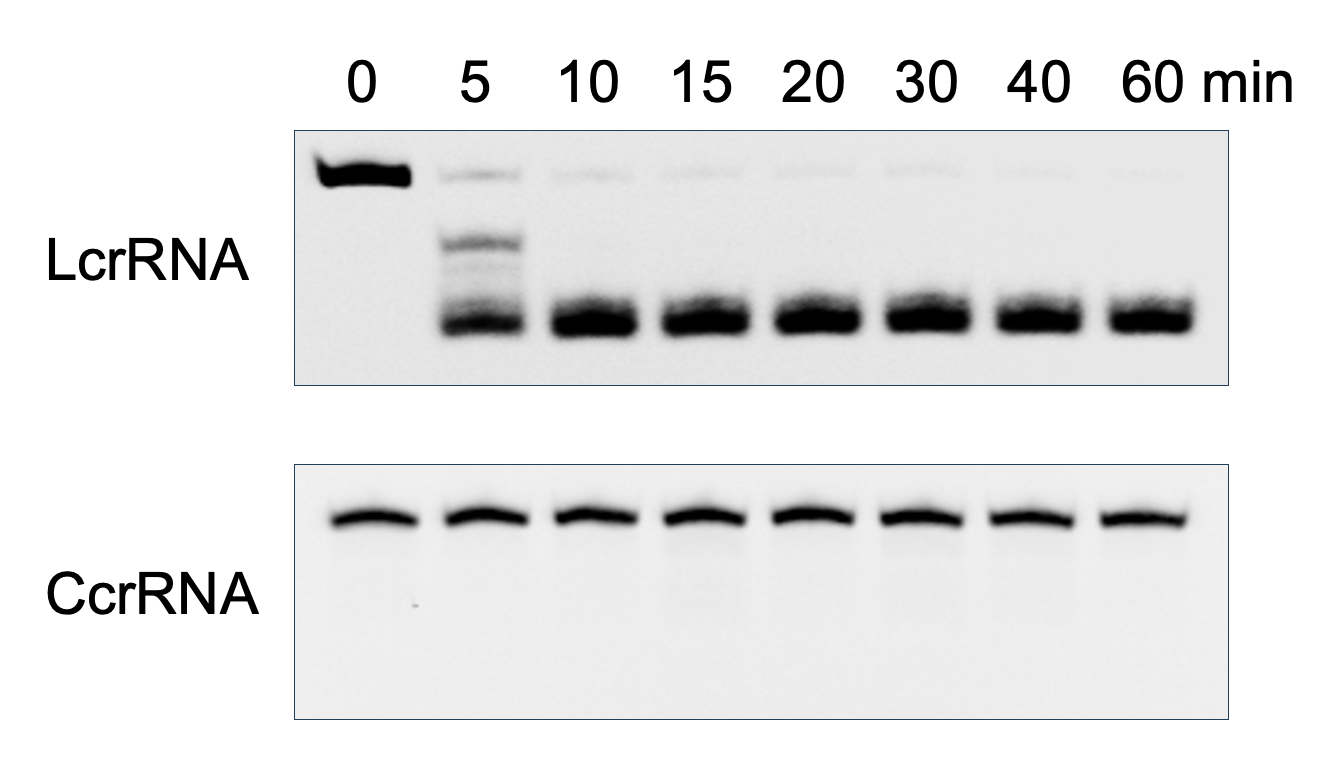


**Figure S4.** Comparison of exonuclease stability between circular crRNA (CcrRNA) and linear crRNA (LcrRNA). 1 µL of 10 µM crRNA samples were incubated at 37℃ for various time points in a 10 µL reaction containing RNase R. The reaction products were analyzed by denaturing urea-PAGE. The experiments were conducted in three technical replicates.


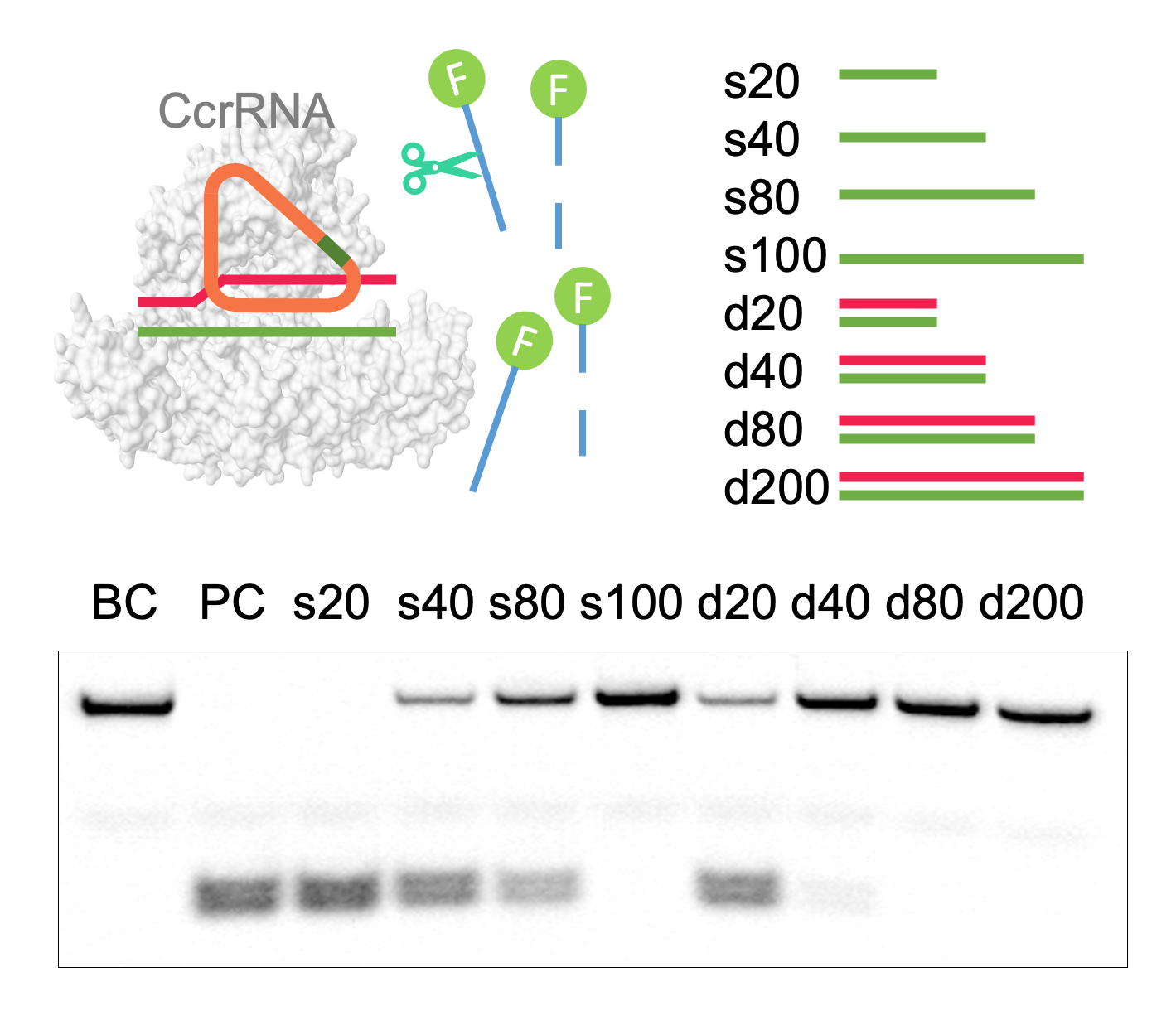


**Figure S5.** Activation of Cas12a–CcrRNA complex trans-cleavage activity by different DNA substrates. In the standard Cas12a trans-cleavage activation assay, circular crRNA (CcrRNA) replaced linear crRNA to assess the activation efficiency of Cas12a trans-cleavage by various DNA substrates (described in the Methods section). The extent of reporter molecule degradation was analyzed by denaturing urea-PAGE. BC: FAM-ssDNA as a blank control; PC: positive control (linear crRNA-DNA activates Cas12a trans-cleavage); s: single strand; d: double strand. The experiments were conducted in three technical replicates.

**
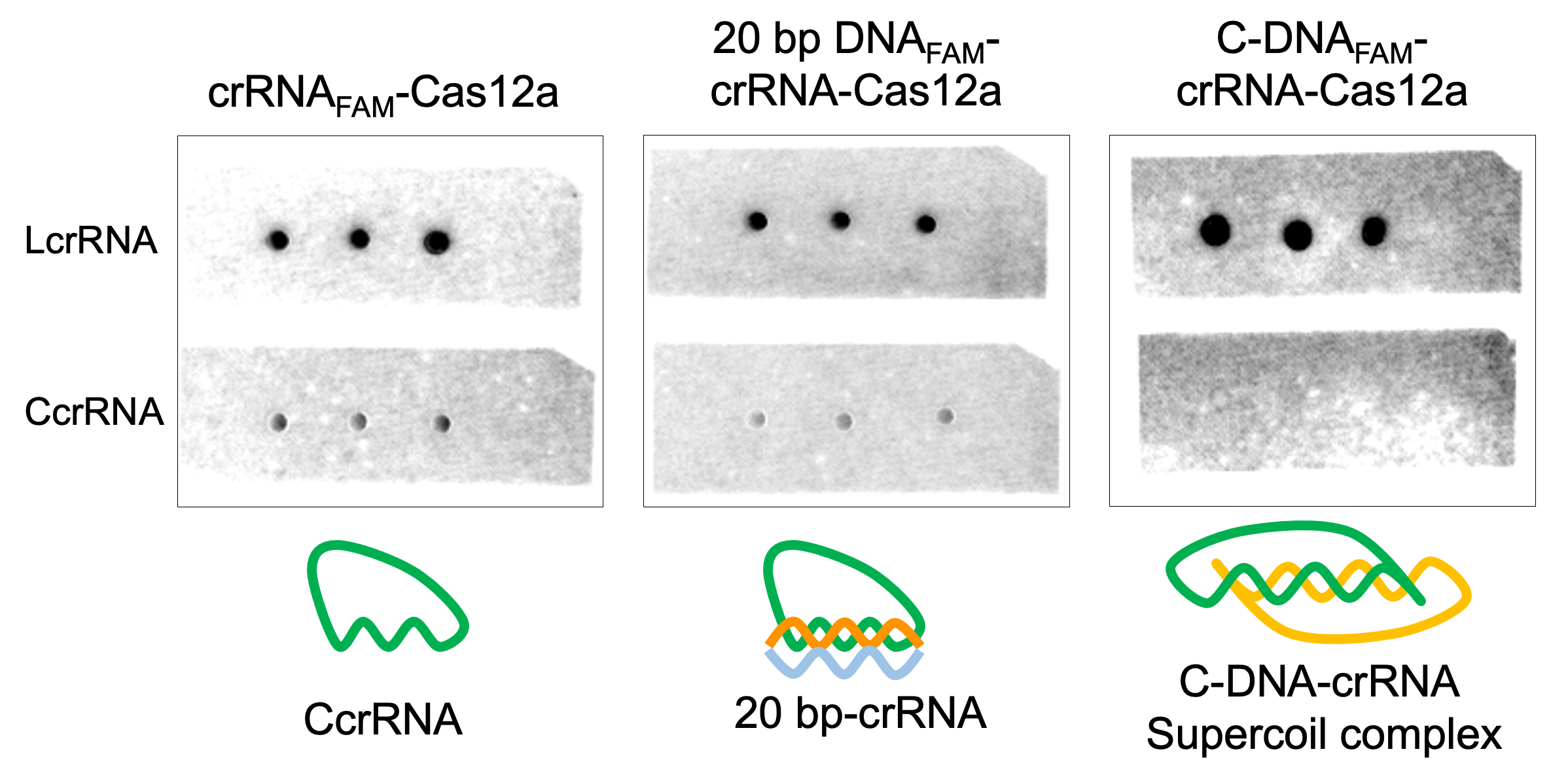
**

**Figure S6.** Dot blot assay comparing binding affinities of different crRNAs and their complexes with various DNA substrates to Cas12a. linear crRNA (LcrRNA) and circular crRNA (CcrRNA) were individually incubated with Cas12a protein. Short double-stranded DNA (20 bp) substrates were pre-incubated with LcrRNA or CcrRNA before binding to Cas12a. Similarly, circular single-stranded DNA (ssDNA) substrates were incubated with LcrRNA or CcrRNA prior to Cas12a binding. Binding affinities were assessed by comparing the fluorescence intensities of FAM-labeled complexes. The experiments were conducted in three technical replicates.


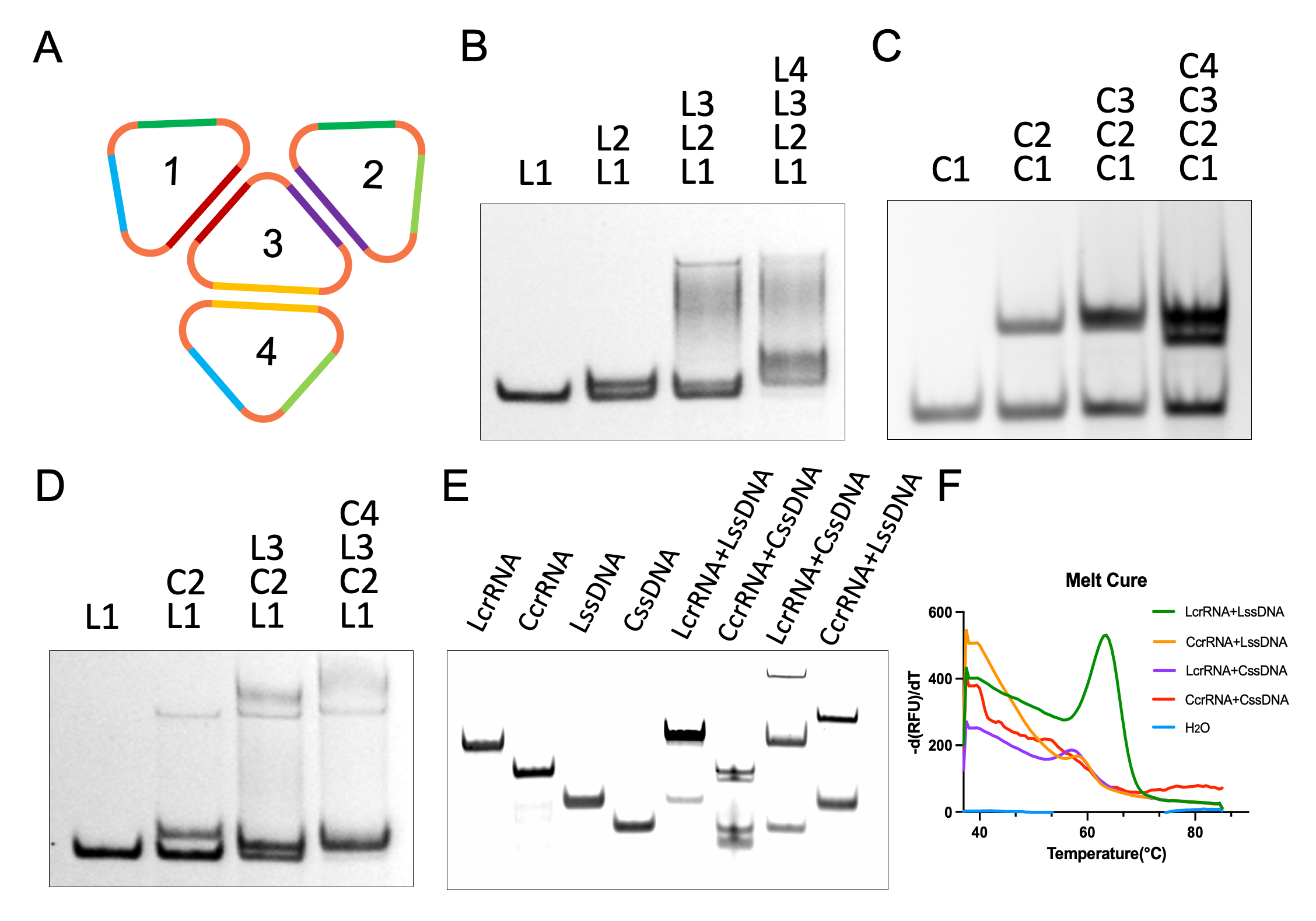


**Figure S7.** Analysis of DNA/RNA higher-order topology formation by complementary base-pairing of circular single-stranded DNA. (A) Schematic illustration of a DNA tetrahedron formed by four complementary circular single-stranded DNA strands. (B) Product analysis of various combinations of four linear single-stranded DNA strands. (C) Product analysis of different combinations of four circular single-stranded DNA strands. (D) Product analysis of complexes formed by circular single-stranded DNA combined with linear single-stranded DNA strands. L: linear; C: circular. (E) Native gel electrophoresis (non-denaturing PAGE) analysis demonstrating the formation of higher-order assemblies between linear/circular crRNA and ssDNA substrates. LcrRNA, linear crRNA; CcrRNA, circular crRNA; LssDNA, linear single-stranded DNA; CssDNA, circular single-stranded DNA. (F) Melting curve analysis of complexes formed between different crRNAs and ssDNA substrates. The experiments were conducted in three technical replicates.


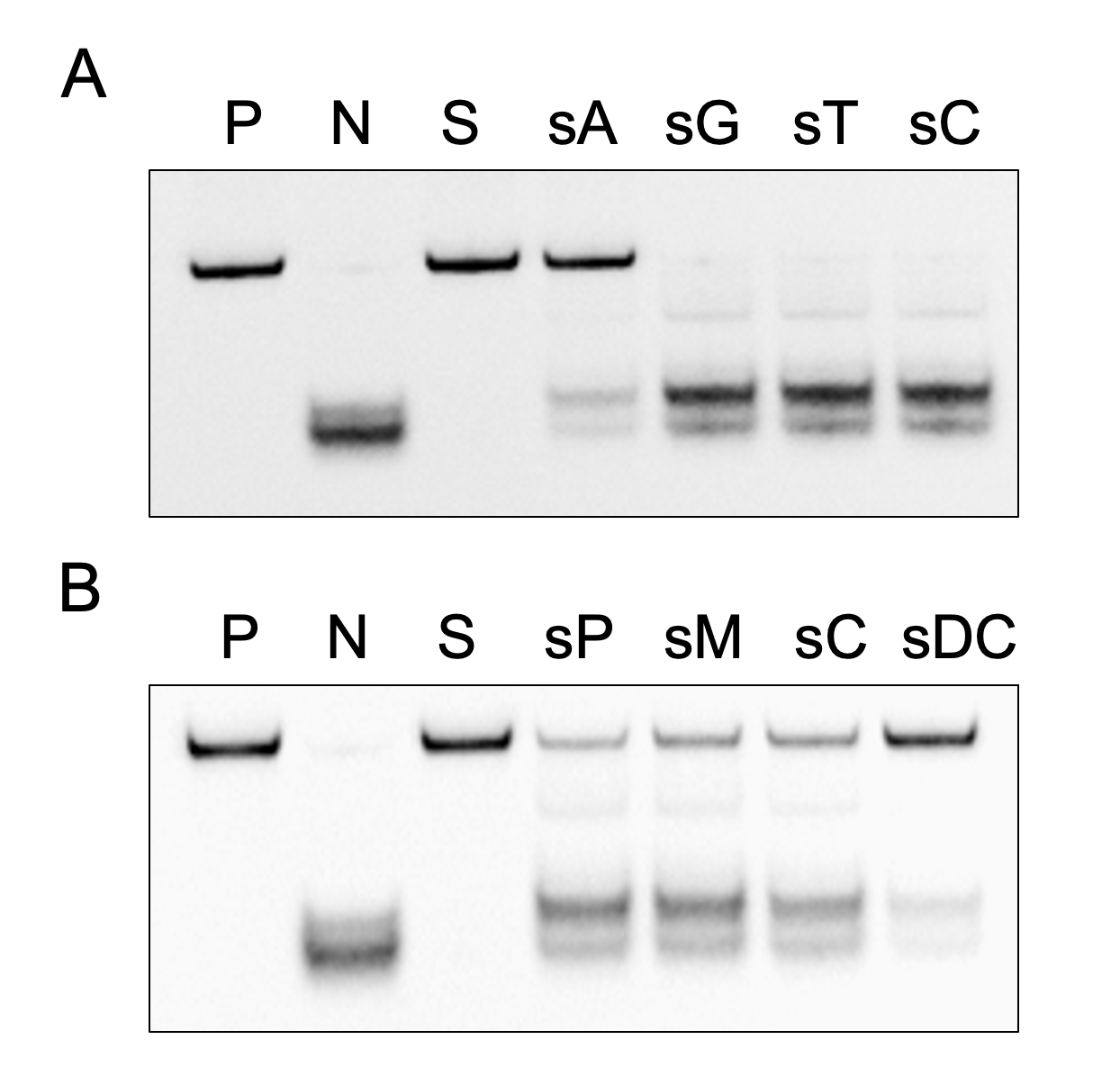


**Figure S8.** Activation of Cas12a trans-cleavage activity by phosphorothioate-modified DNA substrates. (A) Comparison of Cas12a trans-cleavage activation by DNA substrates containing different phosphorothioate modifications. P: ssDNA probe; N: unmodified natural DNA substrate; S: fully phosphorothioate-modified DNA substrate; sA: phosphorothioate-modified dAMP; sG: phosphorothioate-modified dGMP; sT: phosphorothioate-modified dTMP; sC: phosphorothioate-modified dCMP. (B) Comparison of Cas12a trans-cleavage activation by DNA substrates with phosphorothioate modifications at different regions. P: ssDNA probe; N: unmodified natural DNA substrate; S: fully phosphorothioate-modified DNA substrate; sP: phosphorothioate modification at the PAM sequence; sM: phosphorothioate modification at the middle sequence; sC: phosphorothioate modification at the complementary strand cleavage site; sDC: phosphorothioate modification at dual cleavage sites. The experiments were conducted in three technical replicates.


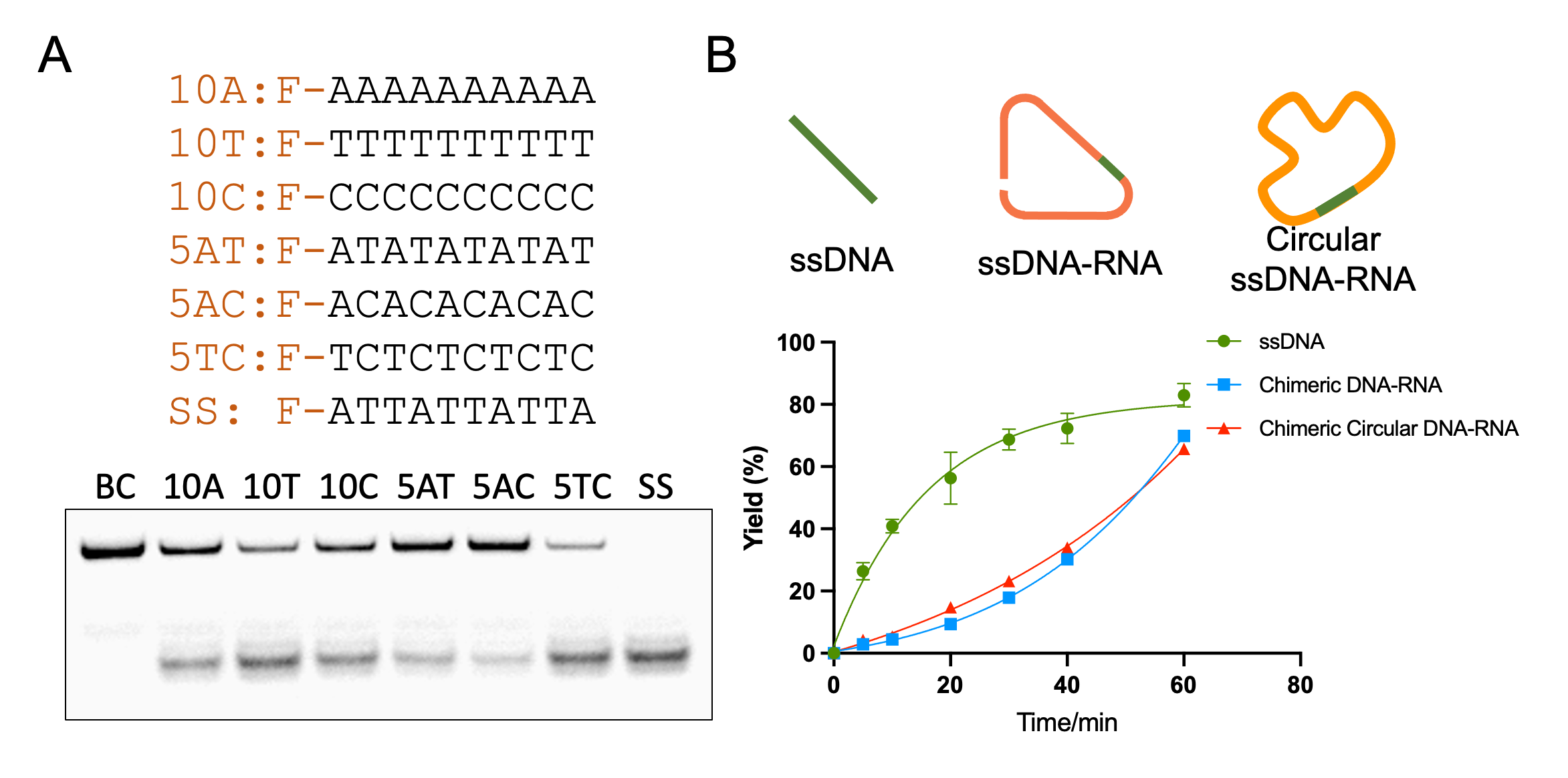


**Figure S9.** Comparison of Cas12a trans-cleavage activity on single-stranded signal molecules with different sequences. Sequence information and denaturing Urea-PAGE analysis of reaction products are shown. P: 10A used as the blank control. (B) Kinetic analysis of Cas12a collateral cleavage on linear DNA, DNA-RNA, and circular DNA-RNA substrates. Reactions were performed with Cas12a (50 nM), crRNA (50 nM), and an ssDNA target (50 nM). After incubation at 37 °C for the indicated times, substrates and products were analyzed by denaturing gel electrophoresis. Data are presented as mean ± SD from three independent experiments (n = 3).


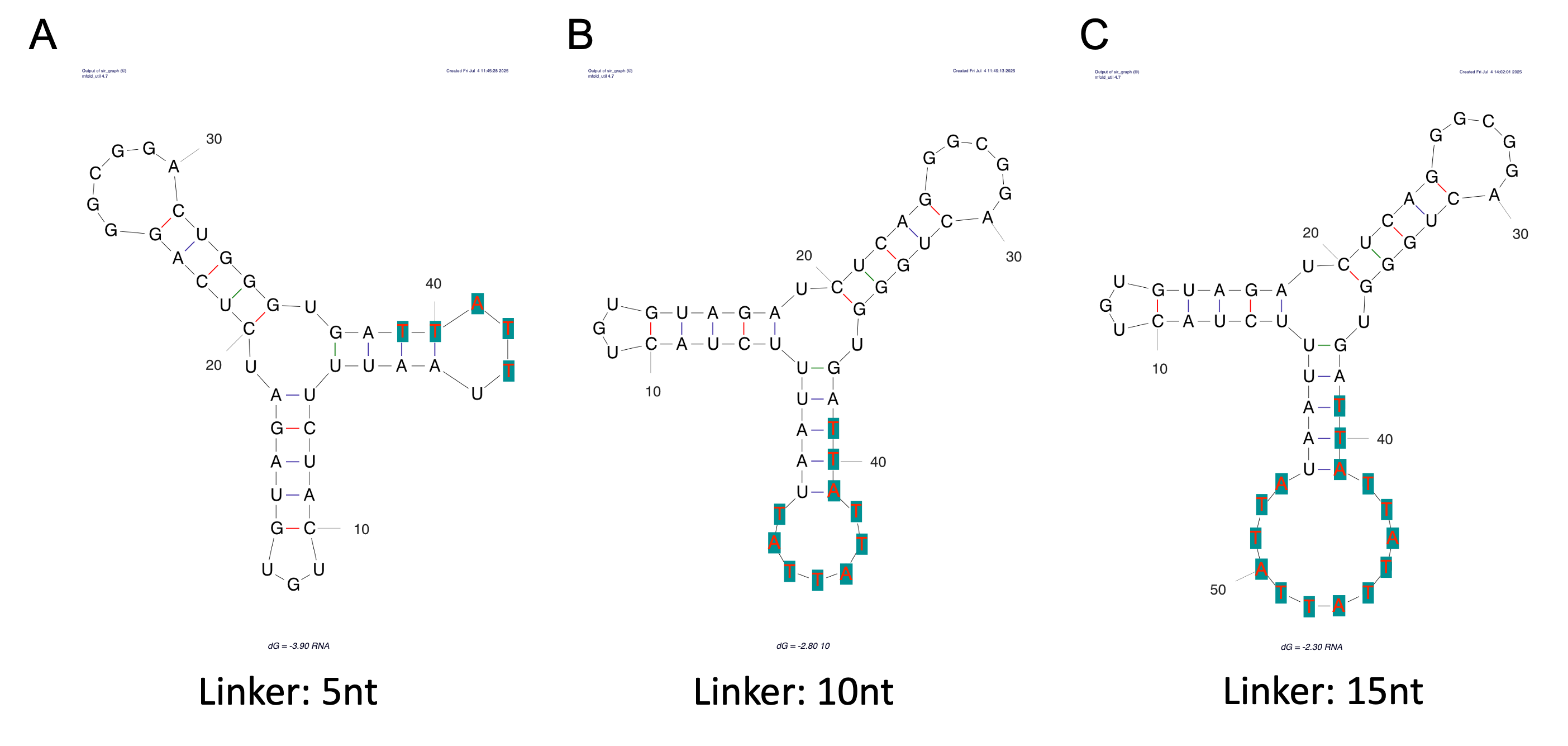


**Figure S10.** Secondary structure analysis of chimeric circular crRNAs with varying DNA segment lengths (5–15 nt) using UNAfold software. (A-C) Predicted secondary structures for crRNAs containing 5 nt, 10 nt, and 15 nt DNA segments, respectively.


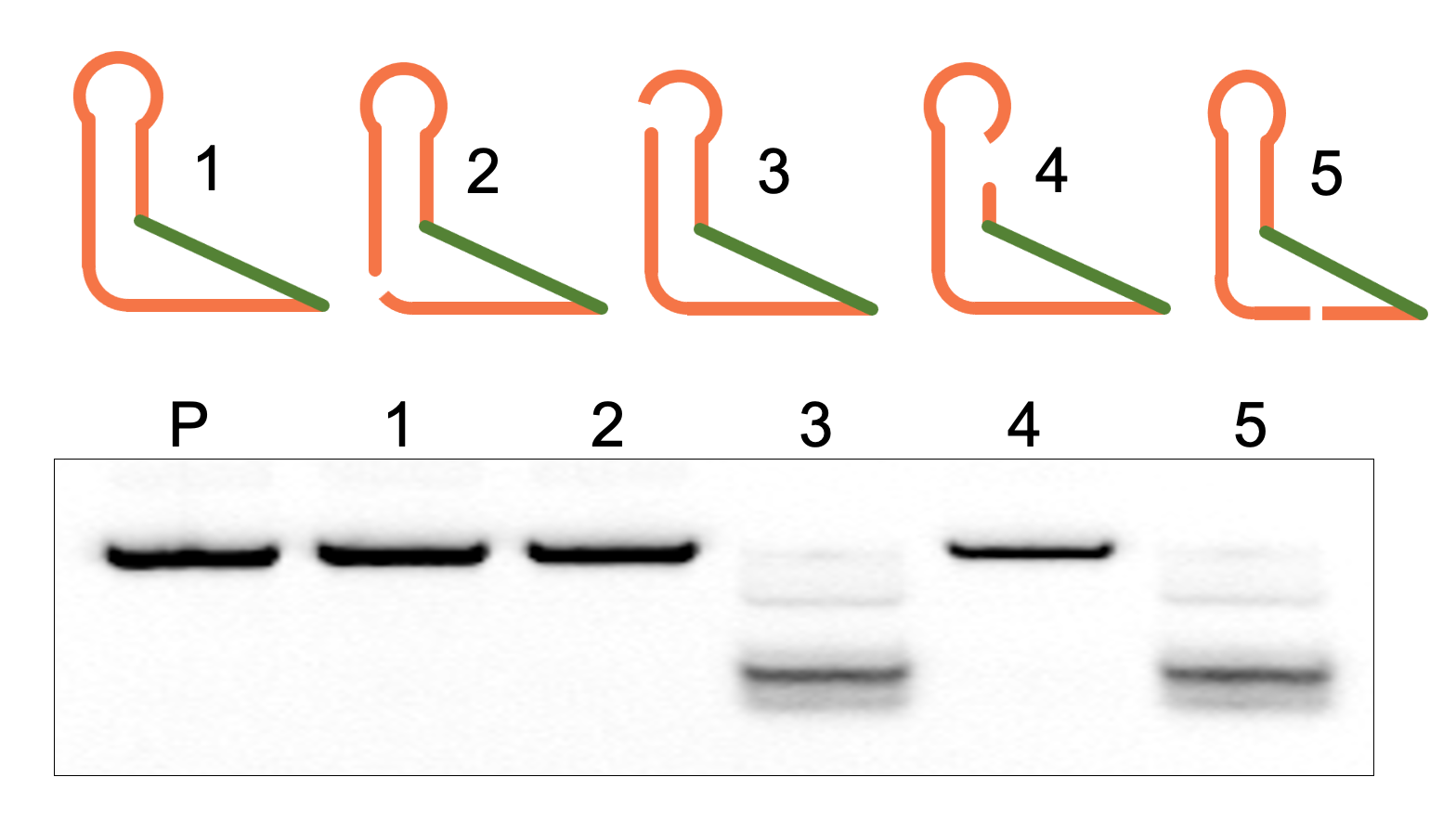


**Figure S11.** Analysis of Cas12a trans-cleavage activity activated by linear crRNA substrates with different circularization points. P: blank control; 1: chimeric circular crRNA; 2: circularization site at the 5′ end of the crRNA spacer region; 3: circularization site in the middle of the crRNA scaffold loop; 4: circularization site in the middle of the crRNA scaffold stem; 5: circularization site in the middle of the crRNA spacer region. The experiments were conducted in three technical replicates.


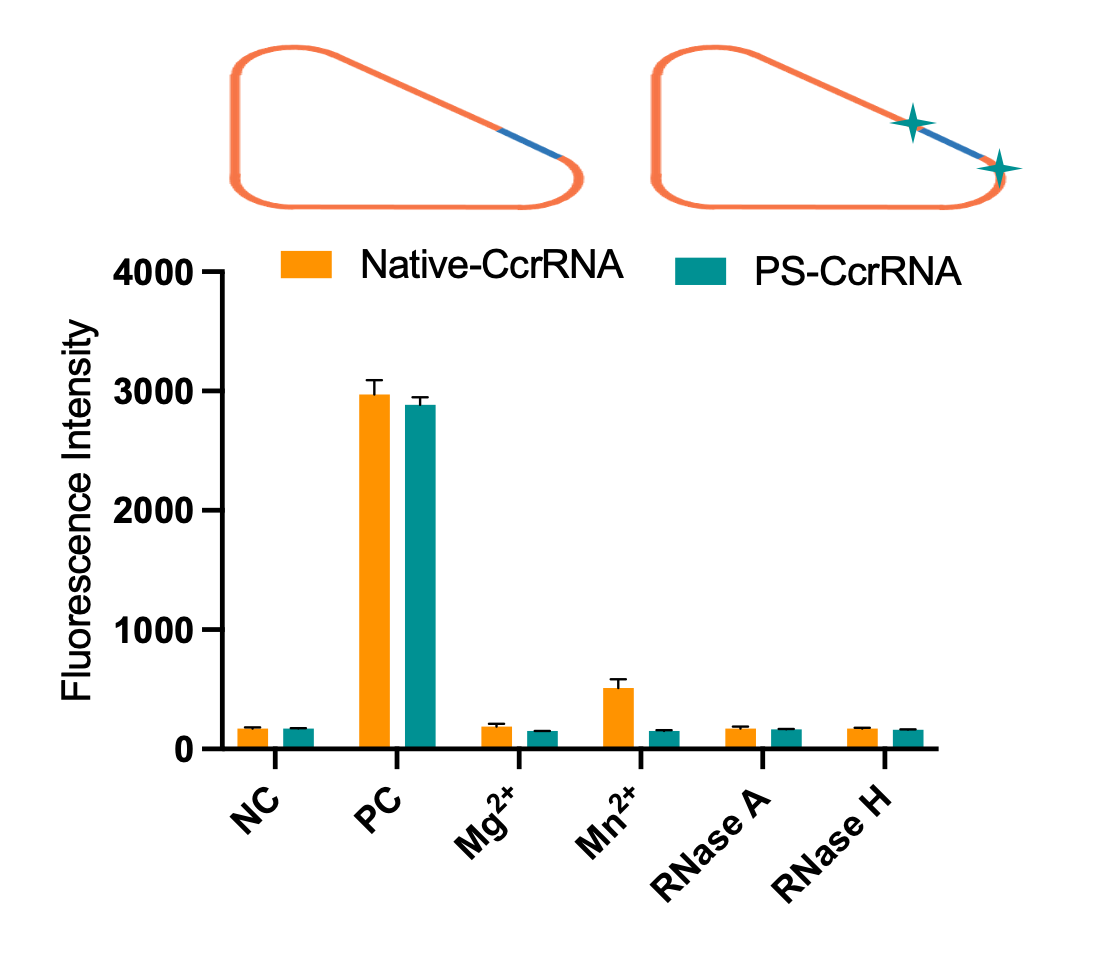


**Figure S12.** Stability analysis of phosphorothioate-modified chimeric circular crRNAs via Cas12a trans-cleavage activation. Phosphorothioate modifications were introduced at the 5′ and 3′ bases of the DNA segment (see Supplementary Table 1). NC: untreated modified and unmodified chimeric circular crRNAs; PC: DNase I-treated modified and unmodified crRNAs, followed by denaturation at 95°C for 10 minutes to inactivate DNase I. Mg²⁺: modified and unmodified crRNAs incubated with 10 mM Mg²⁺ at 95°C for 10 minutes; Mn²⁺: modified and unmodified crRNAs incubated with 10 mM Mn²⁺ at 95°C for 10 minutes; RNase A: treated with 0.1 U/μL RNase A; RNase H: treated with 0.1 U/μL RNase H. Following treatments, crRNAs were added to the standard Cas12a trans-cleavage activation assay containing a FAM-BHQ1 reporter molecule, and fluorescence intensity was measured. A four-pointed star denotes phosphorothioate modification of the nucleic acid phosphate backbone. Data are presented as mean ± SD from three independent experiments (n = 3).


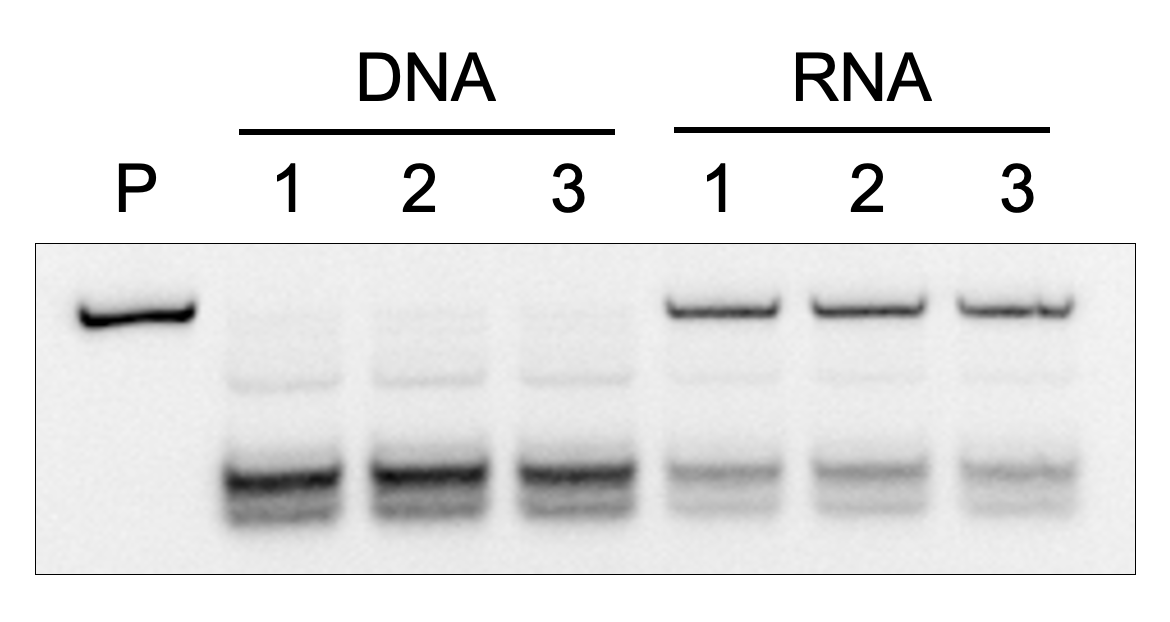


**Figure S13.** Activation of Cas12a trans-cleavage activity by RNA substrates. P: blank control. DNA and RNA substrates were each tested in triplicate.


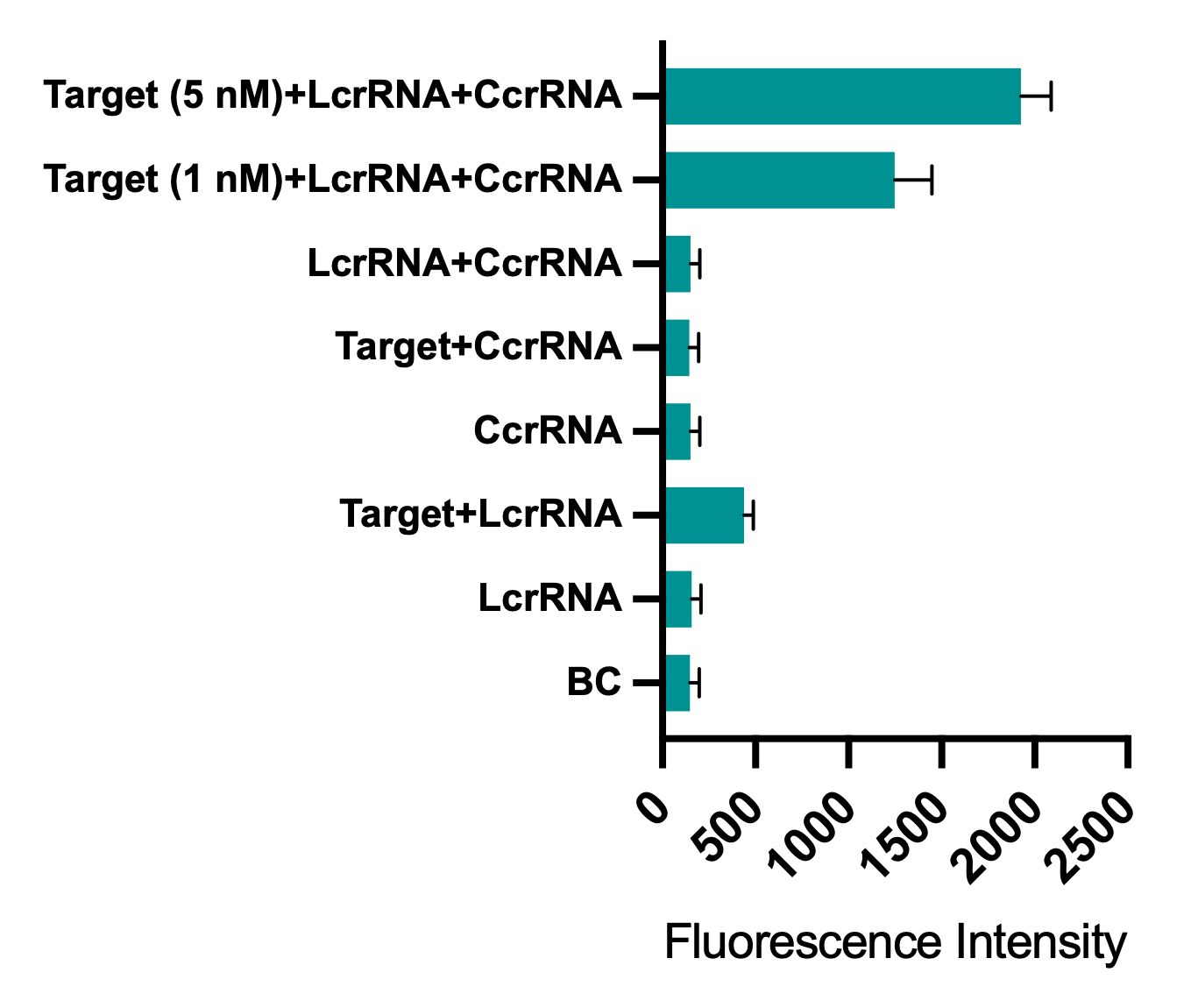


**Figure S14.** TopCas system activation by RNA substrates. Data are presented as mean ± SD from three independent experiments (n = 3).


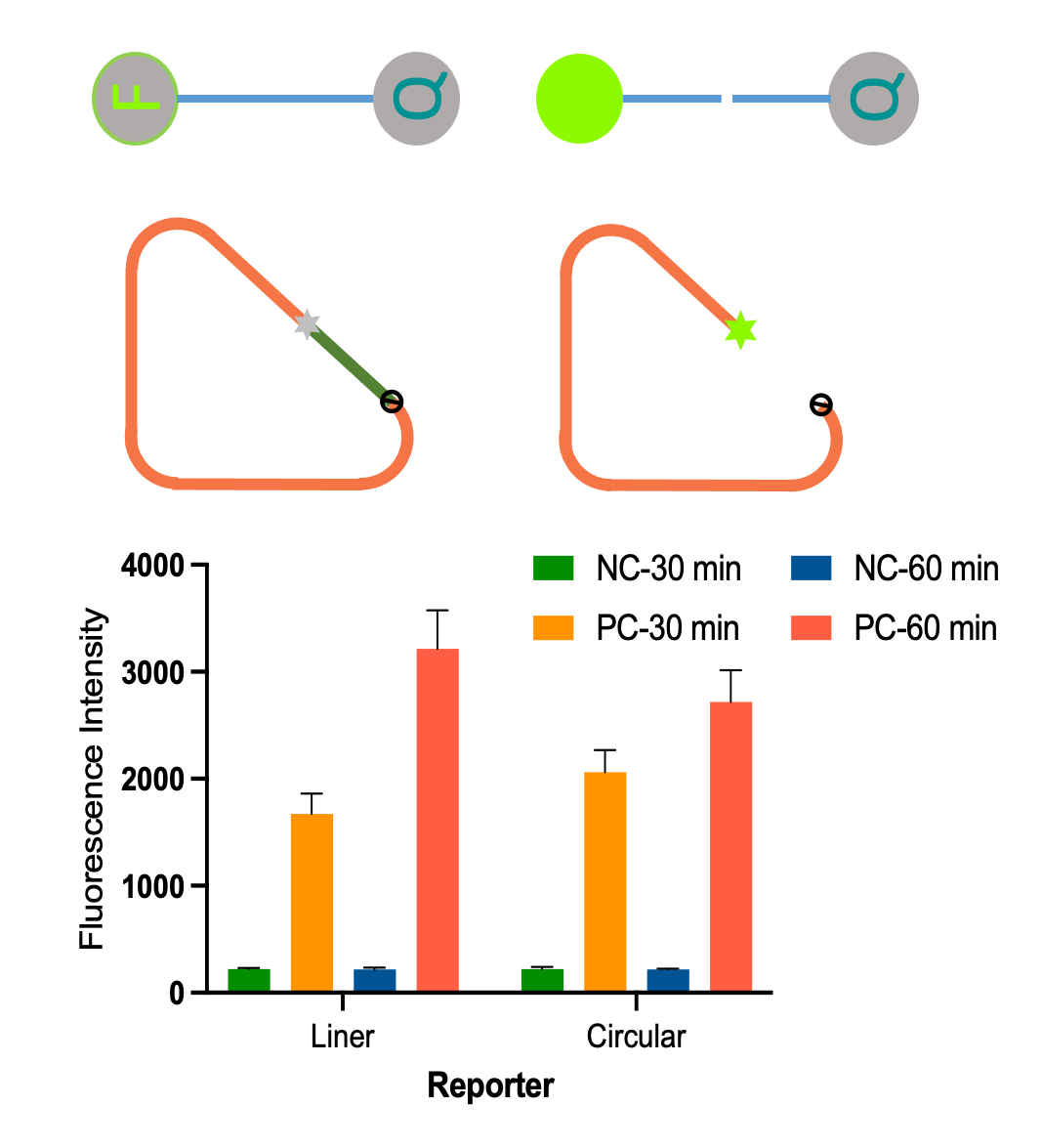


**Figure S15.** Different types of fluorescent molecular probes used in the TopCas system. Linear ssDNA probes labeled with fluorophore and quencher groups at opposite ends, and chimeric circular crRNA probes with fluorophore and quencher modifications at both ends of the DNA segment. Data are presented as mean ± SD from three independent experiments (n = 3).


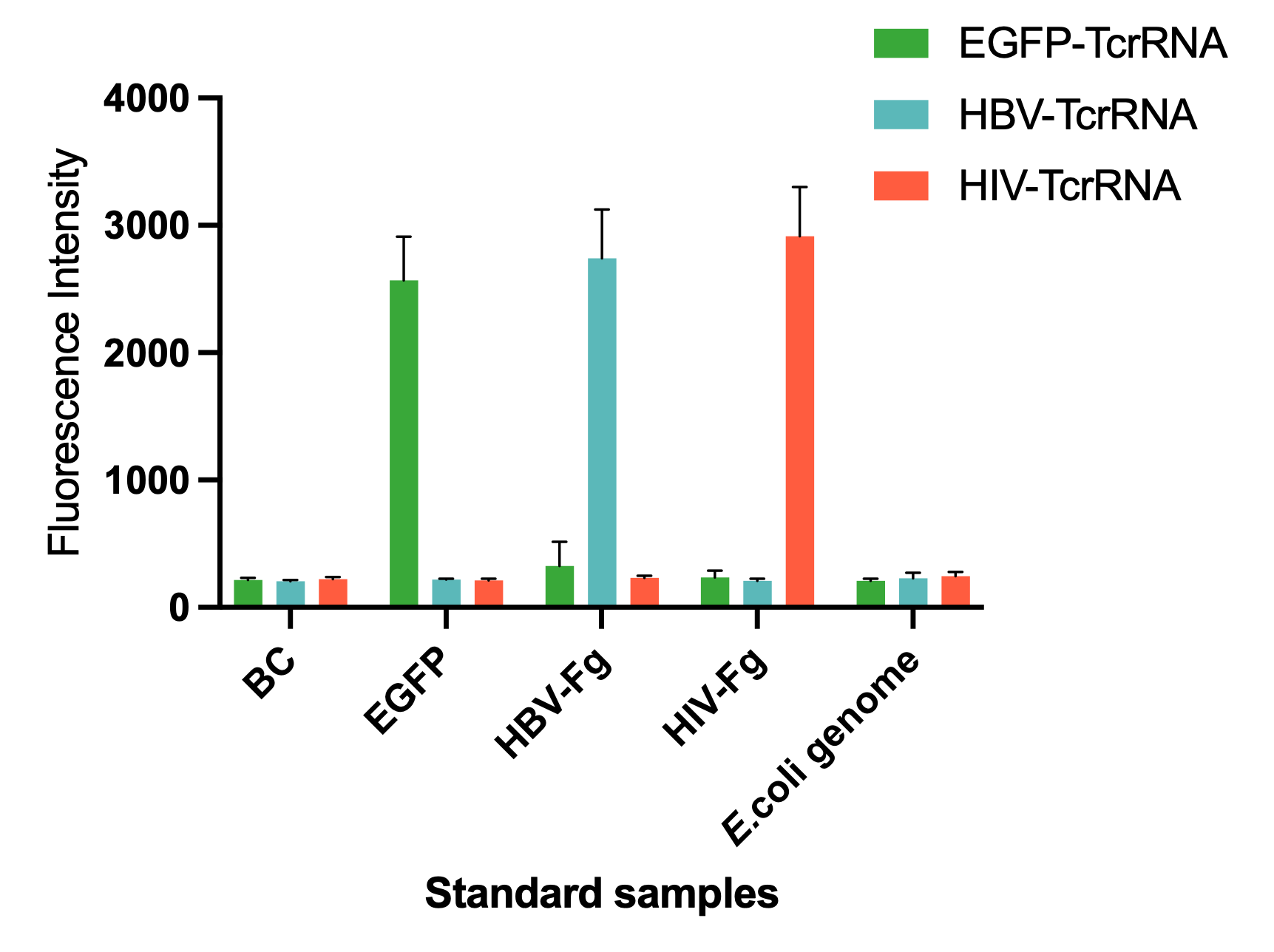


**Figure S16.** Standard target nucleic acid molecules were detected by TopCas. The cross-detection assays using different targets and TcrRNA. BC: blank control, *E*. coli genome as negative control with background DNA. Data are presented as mean ± SD from three independent experiments (n = 3).


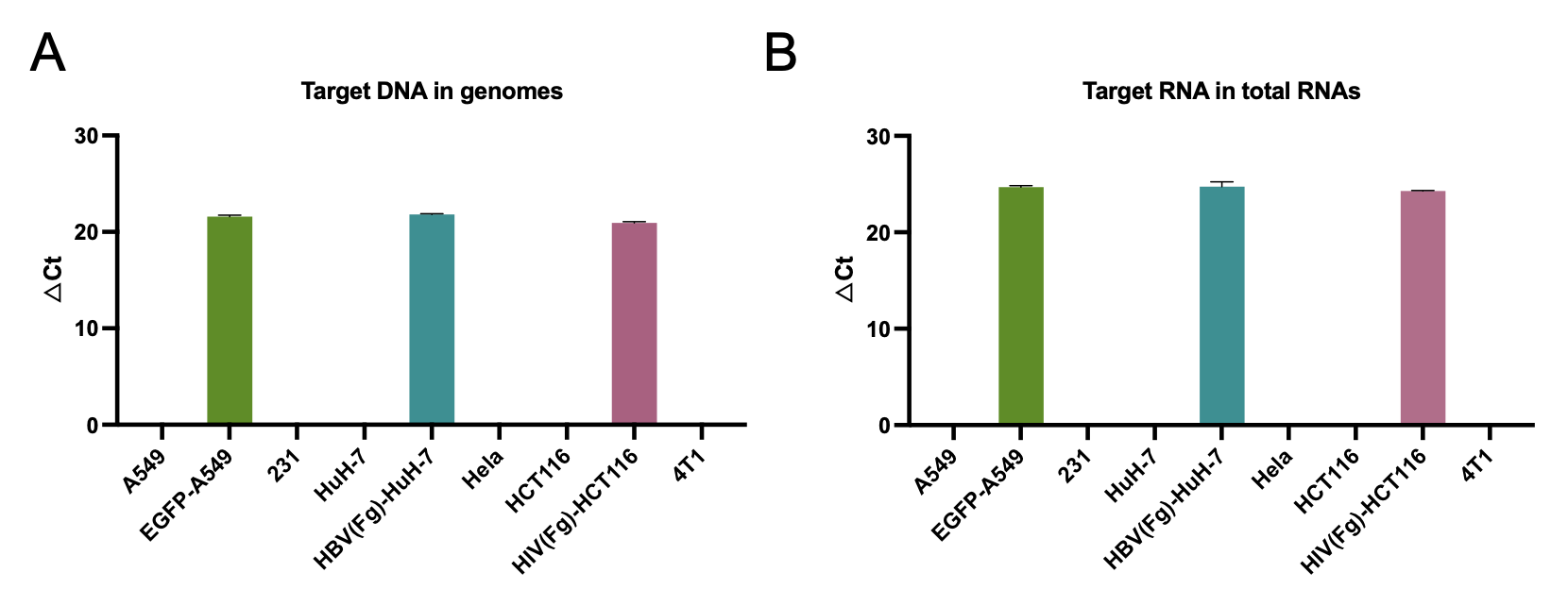


**Figure S17.** qPCR detection of target nucleic acids in complex background matrices. (A) Detection of target DNA in complex mixtures by qPCR; targets include EGFP, HBV(Fg), and HIV(Fg), with genomic DNA serving as background. (B) Detection of target RNA in complex mixtures by RT–qPCR; targets include EGFP, HBV(Fg), and HIV(Fg), with total cellular RNA serving as background. Data are presented as mean ± SD from three independent experiments (n = 3).


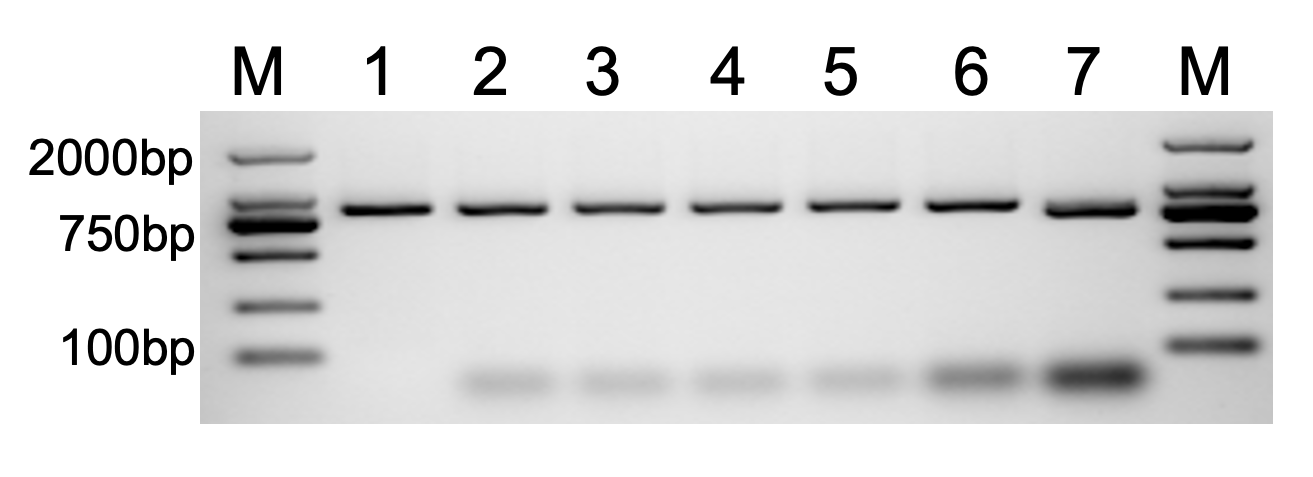


**Figure S18.** Conditional gene editing using the TopCas system targeting the second editing site of EGFP. Cis-cleavage activity of the TopCas system on target DNA with various component combinations. M: DNA marker; 1: target DNA; 2: TcrRNA; 3: trigger DNA + TcrRNA; 4: CcrRNA + circular ssDNA substrate (C-ssDNA); 5: trigger DNA + CcrRNA + C-ssDNA; 6: TcrRNA + CcrRNA + C-ssDNA; 7: trigger DNA + TcrRNA + CcrRNA + C-ssDNA. All reactions (1–7) contained Cas12a protein, target DNA and standard reaction buffer. The experiments were conducted in three technical replicates.


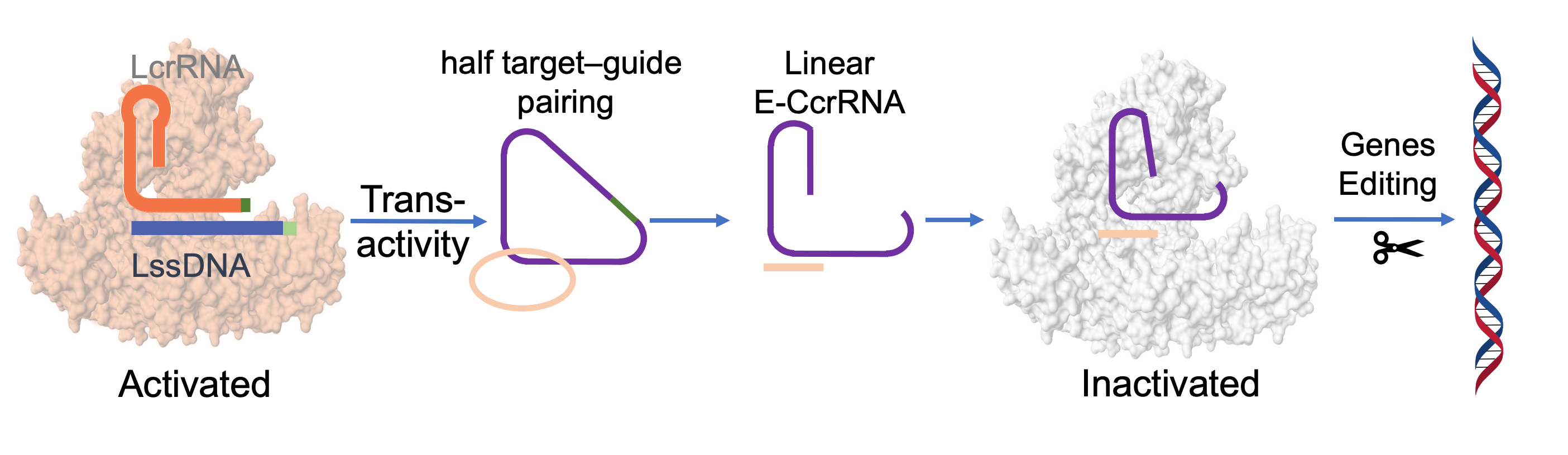


**Figure S19.** Detailed schematic of conditional gene editing enabled by TopCas.
